# Atomic Force Microscopy for Cross‐Disciplinary Materials Research

**DOI:** 10.1002/smtd.202500514

**Published:** 2025-06-23

**Authors:** Soyun Joo, Seongmun Eom, Youngwoo Choi, Uichang Jeong, Yoonhan Cho, WonJeong Yu, Kunwoo Park, Seungbum Hong

**Affiliations:** ^1^ Department of Materials Science and Engineering Korea Advanced Institute of Science and Technology (KAIST) Daejeon 34141 Republic of Korea; ^2^ KAIST Institute for NanoCentury (KINC) Korea Advanced Institute of Science and Technology (KAIST) Daejeon 34141 Republic of Korea

**Keywords:** atomic force microscopy, experimental optimization, microscopy techniques, quantitative data analysis, surface property mapping

## Abstract

While microscopy remains the primary method for verifying material structures, recent technological advancements have both enabled and necessitated the analysis of ever‐finer details. Unlike scanning electron microscopy (SEM) and transmission electron microscopy (TEM), atomic force microscopy (AFM) provides unique capabilities beyond visualization, mapping surface properties through precisely controlled physical interactions between the probe and sample. In materials research specifically, AFM has become indispensable for characterizing mechanical, electrical, chemical, and magnetic properties at the nanoscale with exceptional spatial resolution. With ongoing technological progress and the expansion of specialized imaging modes, AFM enables cross‐disciplinary collaboration across various materials science domains, from electronic materials to energy storage systems. However, its effective implementation is often challenged by the technical complexity and varied domain expertise among collaborators. This review examines critical considerations in AFM‐based research, from experimental protocols to quantitative data analysis. Validated approaches for measurement optimization are presented to ensure reproducibility and support successful cross‐disciplinary AFM implementation. The review includes detailed implementation guidance for advanced AFM methodologies and comprehensive case studies spanning diverse material systems. By providing theoretical foundations and practical guidance, this review aims to facilitate more effective collaboration across disciplines, ultimately advancing the use of AFM in complex, multi‐faceted research.

## Graphical abstract

This figure demonstrates the broad applicability of atomic force microscopy (AFM) across interdisciplinary materials research. Interlocking puzzle pieces illustrate how AFM integrates with complementary analytical techniques to provide comprehensive material characterization. The central cube represents the target system, each face corresponding to distinct material properties. In such dynamic, the AFM probe works as a fundamental tool to investigate diverse nanoscale phenomena, elucidating complex material behaviors.

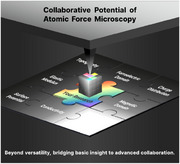

## 1 Introduction

The nanoscale characterization of materials has become increasingly critical across diverse scientific disciplines, from emerging quantum materials to advanced biological systems. Among various nanoscale characterization techniques, atomic force microscopy (AFM) stands out for its versatility in providing topographical information and insights into mechanical, electrical, and chemical properties at the nanoscale. Since its invention by Binnig, Quate, and Gerber in 1986,^[^ ^1^ ^]^ AFM has evolved from a specialized surface imaging tool to an indispensable platform for interdisciplinary research collaboration, particularly in materials science and engineering.

The journey of AFM from its origins to its current state reflects the broader evolution of nanoscale research. Initially designed to overcome the limitations of scanning tunneling microscopy (STM), particularly its restriction to conductive samples, AFM opened new possibilities for investigating various materials under multiple environmental conditions. The technique's fundamental principle—using a microscopic cantilever with an ultra‐sharp tip to interact with material surfaces—has remained unchanged. However, its applications have expanded dramatically through technological advances and innovative measurement modes.

Despite its widespread adoption, the complexity of AFM techniques has created a significant gap between specialists and collaborators from other fields. This gap manifests in several critical challenges that often impede successful collaboration. Surface preparation requirements vary significantly depending on the measurement type and can be particularly demanding for specialized modes such as piezoresponse force microscopy (PFM) or Kelvin probe force microscopy (KPFM). Environmental factors, including humidity, temperature, and electromagnetic interference, can significantly impact measurement quality, yet non‐specialists often underappreciate their effects.

Mode selection presents another substantial challenge. Modern AFM systems offer multiple operational modes, each with its strengths and limitations. Specialized modes for probing specific properties require careful consideration of measurement parameters and environmental conditions. The choice of mode significantly affects not only the quality of results but also the type of information obtained, the achievable resolution, and the sample's integrity during measurement.

Data interpretation represents perhaps the subtlest yet most intricate challenge in AFM‐based collaboration. AFM measurements typically involve complex convolutions of multiple physical effects, challenging distinguishing genuine signals from artifacts. Tip‐sample interaction effects, calibration procedures, and scaling considerations are critical in obtaining reliable quantitative measurements. Moreover, statistical analysis of AFM data requires careful consideration of measurement uncertainties and sampling strategies.

While multiple, comprehensive technical reviews exist on specific AFM techniques,^[^ ^2^, ^3^, ^4^, ^5^, ^6^, ^7^, ^8^, ^9^ ^]^ there is a notable absence of guides addressing the practical aspects of AFM‐based collaboration in materials research. This gap becomes particularly evident in cross‐disciplinary projects, where researchers from different fields must establish common ground for effective communication and project execution. Integrating AFM with complementary characterization techniques further complicates these challenges, requiring careful coordination of sample preparation, measurement sequences, and data analysis protocols.

This comprehensive review is written to bridge these gaps by providing both theoretical understanding and practical insights for successful AFM‐based collaboration. Beginning with fundamental principles, we explain the key components and operational mechanisms of AFM systems, emphasizing aspects critical for non‐specialists (Section 2). Subsequent sections address practical considerations in experiment preparation, mode selection, and measurement optimization (Section 3). Particular attention is given to data analysis and interpretation protocols, including detailed discussions of image processing methods and statistical analysis strategies (Section 4).

The review also establishes a framework for effective collaboration, drawing from our laboratory's extensive experience in cross‐disciplinary research. We highlight the complementary strengths of AFM and other techniques, offering guidance on their distinctions, along with protocols for communication, project planning, and resource‐sharing (Section 5). Case studies from various materials research fields illustrate successful collaboration approaches while highlighting potential pitfalls and their solutions (Section 6).

Although our primary focus is on materials science applications, where the diversity of material classes and properties demands specialized approaches to AFM characterization, many principles and methodologies discussed are transferable to other fields where AFM is employed. The fundamental challenges of interdisciplinary collaboration, technical communication, and data interpretation remain relevant regardless of the specific application domain, making this review a valuable resource for researchers across scientific disciplines engaging with AFM technology.

By addressing both technical and collaborative aspects of AFM research, this review serves as a bridge between specialists and collaborators, enabling more effective and productive research partnerships. Whether you are a materials scientist conducting surface characterization, a biologist investigating cellular mechanics, or an AFM specialist aiming to improve collaborative outcomes, this review provides the framework for successful interdisciplinary research using AFM. Through careful attention to theoretical foundations and practical implementation, we aim to facilitate the broader adoption of advanced AFM techniques while ensuring reliable and reproducible results in collaborative research settings.

## 2 A Brief Overview of AFM

AFM is based on the precise interactions between a nanoscale tip and a sample surface, allowing for high‐resolution imaging and characterization of surface properties at the nanoscale. At its core, AFM utilizes a cantilever with an ultra‐sharp tip that interacts with the sample through various atomic forces. These interactions are translated into measurable signals, facilitating detailed analysis of surface topography, mechanical properties, and functional behaviors. In the following sections, we discuss how the unique configuration of AFM enables these measurements at resolutions unattainable with other techniques. Given the diverse backgrounds of AFM users, a brief yet technical discussion is provided to establish a foundation for designing experiments and accurately interpreting their results.

### 2.1 How AFM Works

#### 2.1.1 Components of AFM

**Figure 1:**
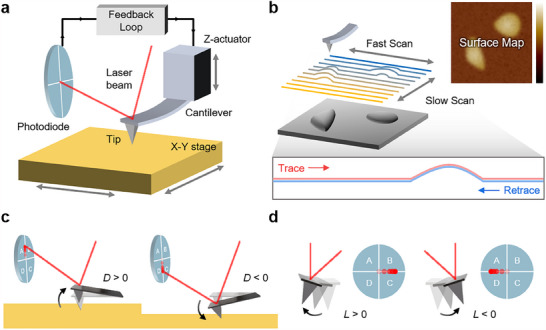
Working Principle of AFM. a) The tip scans the sample surface while a laser beam reflects off the back of the cantilever onto a photodiode, recording tip movements. A feedback loop dynamically adjusts the Z‐actuator to maintain consistent distance between the tip and sample. b) The tip follows a line‐by‐line raster pattern, generating a 3D surface map of the scanned area. Each fast scan line involves two passes: a trace and a retrace. c) Vertical bending and d) lateral twisting movements of the cantilever are converted into electrical signals through the beam‐bouncing method.

The core functionality of an AFM system relies on its key components, which operate in concert to achieve nanoscale precision (**Figure** **1**a). The primary element is a flexible cantilever with an ultra‐sharp tip, typically a few nanometers of radius at its apex. As the tip interacts with the sample surface, a laser beam reflects off the cantilever's back onto a position‐sensitive photodiode detector (PSPD), tracking deflections that occur as a result of variations in surface property. Both vertical and lateral movements are recorded to generate images of the surface, where each line profile in the fast scan direction is created by moving the tip back and forth (trace and retrace) across the surface. A series of these line profiles, captured at incremented positions in the slow scan direction, is combined to create a detailed, three‐dimensional surface map (Figure 1b).

Meanwhile, the movement control system for positioning the tip on the desired location includes piezoelectric actuators for precise movement in three dimensions: two for lateral (X‐Y) scanning across the surface (with ranges from 30 to 100 micrometers limits, depending on the AFM model), and one for vertical (Z) adjustment with typical ranges around 10 micrometers. A sophisticated closed‐loop feedback system ensures precise control over tip‐sample interactions at the picometer scale, while the Z‐actuator continuously adjusts the tip's height based on the feedback signals.

#### 2.1.2 Signal Detection

The beam‐bouncing method embodies the signal detection technique used in AFM (Figure 1c,d). By comparing the intensity of the laser beam detected at each quadrant (*I_A_*, *I_B_*, *I_C_*, and *I_D_*), the photodiode accurately captures both the vertical and lateral displacements. Vertical displacements contain topographical height information, while lateral displacements correspond to torsional movements and frictional information. The deflection signal *D*, representing the vertical displacement, is calculated using the difference in intensities of the upper and lower sets of quadrants:

(1) $$D=\left(I_{A}+I_{B}\right)-\left(I_{C}+I_{D}\right)$$

When the cantilever is at rest, the photodiode should be calibrated to center the reflected laser beam (*D = 0*). If the cantilever bends upwards, the reflected beam moves up, increasing intensities detected at the upper half of the photodiode compared to the lower half, resulting in a positive deflection signal (*D* > 0) (Figure 1c). Conversely, if the cantilever bends downward, the beam moves down, leading to a negative deflection signal (*D* < 0).

Lateral deflections are quantified as follows, indicating torsional movements caused by lateral forces such as friction or shear at the tip‐sample interface (Figure 1d).

(2) $$L=\left(I_{A}+I_{D}\right)-\left(I_{B}+I_{C}\right)$$

In addition to optical beam deflection, alternative cantilever‐deflection sensing methods have been developed to support operation in conditions where optical alignment is impractical or stability requirements are stringent. The qPlus sensor employs a quartz tuning fork with one prong fixed and a tip mounted on the free prong. It uses piezoelectric charge detection to measure oscillatory motion electrically, eliminating the need for a laser or photodiode. The high stiffness of the tuning fork (typically on the order of 2000 N m^−1^)^[^ ^10^ ^]^ enables stable operation at sub‐angstrom amplitudes, reducing susceptibility to jump‐to‐contact and improving spatial resolution. The KolibriSensor, based on a symmetric tuning fork configuration, allows for concurrent detection of frequency shift and tunneling current.^[^ ^11^ ^]^ This facilitates combined AFM and STM measurements, particularly in ultra‐high vacuum or low‐temperature environments where optical access is limited. Both sensors represent self‐sensing detection systems and are used in non‐contact or frequency modulation AFM modes for applications requiring mechanical and electronic signal acquisition with high spatial and force sensitivity.

#### 2.1.3 Surface Tracking Methods

Then how are the collected signals translated into surface information, and how does AFM track the surface? The answer consists of two parts, as AFM can operate in two modes: contact mode (CM) and non‐contact mode (NCM). While these modes are discussed with greater detail in Section 3.1.1, the following provides an overview of how cantilever movements are monitored and translated into surface data.

In CM, the tip maintains direct physical contact with the sample surface, where the cantilever's deflection directly responds to surface topography through repulsive forces. The degree of bending varies based on the magnitude of this repulsive force. To ensure consistent measurement, a reference force known as the setpoint is defined to maintain a constant force and corresponding tip deflection. A feedback system continuously adjusts the Z‐scanner position to maintain a constant deflection (setpoint), allowing the system to map surface features while simultaneously measuring various physical properties through the tip‐sample interaction.

NCM, a dynamic mode in AFM,^[^ ^3^ ^]^ operates through a fundamentally different principle, where the tip oscillates near its resonance frequency above the sample surface without making direct contact. Instead of measuring direct deflection, NCM monitors changes in the oscillation amplitude (amplitude modulation AFM; AM‐AFM) or frequency shift (frequency modulation AFM; FM‐AFM), which vary with the force and force gradient, respectively, between the tip and the sample. The feedback system maintains a constant tip‐sample separation by adjusting the Z‐position to keep the oscillation amplitude and frequency shift at a preset value (setpoint), enabling precise surface mapping through force equilibrium measurements while minimizing lateral forces that could distort the image.

The choice between CM and NCM depends on the physical properties of the sample and the experimental requirements. CM provides direct access to various physical property measurements while maintaining stable surface tracking. However, due to the direct contact involved, the tip can damage soft or fragile samples. In contrast, NCM avoids direct contact, offering superior topographical resolution and non‐invasive characterization, which is particularly valuable for sensitive samples or atomic‐scale imaging. Nevertheless, the larger tip‐surface interaction area of NCM compared to CM complicates achieving high resolution in property mapping, potentially reducing the resolution in such measurements.

### 2.2 What AFM Measures

As explained, surface topography is determined by recording the height of the Z‐scanner, which controls the AFM probe's vertical position during analysis. In addition to the Z‐scanner data, laser signals reflected from the probe's interaction with the sample surface carry a wealth of information. However, the interpretation of these laser‐based signals is highly dependent on the probe's material properties and the specific AFM mode employed. Identical numeral values can convey vastly different meanings under varying conditions. Thus, users need to understand the types of data generated by AFM analysis and their precise meanings.

#### 2.2.1 Height

Height measurement is a fundamental aspect of AFM analysis, providing quantitative visualization of surface morphology by detecting the attractive and repulsive forces between the AFM probe and the material surface. Far more than simply revealing two‐dimensional (2D) micrometer‐scale surface features, this three‐dimensional (3D) data that ranges from the picometer to nanoscale bridges the gap between microscopic surface structures and their influence on macroscopic material properties.

#### 2.2.2 Deflection

In CM topography imaging, deflection serves as an additional data output, representing the bending of the AFM probe as it interacts with the material's surface. While surface morphology can be visualized from variations in deflection with a fixed probe height, the more common method involves maintaining a constant deflection during imaging. This method reduces the risk of probe damage and ensures more reliable data acquisition. Ideally, the deflection signal should remain constant; however, factors such as imperfect contact due to surface roughness,^[^ ^12^, ^13^, ^14^ ^]^ un‐feedbacked probe‐sample interactions, and artifacts from mechanical crosstalks^[^ ^15^, ^16^ ^]^ often introduce fluctuations. These variations primarily reflect the surface's slope, as abrupt height changes can exceed the AFM feedback system's response capability. Consequently, regions with significant deflection changes typically indicate areas of steep surface gradients.

In addition to surface slope, deflection data can reveal other material properties depending on the experimental setup, material characteristics, and probe features. For example, in lateral force microscopy (LFM), the torsional deflection of the cantilever during horizontal scanning is measured to assess lateral forces. This measurement correlates with frictional interactions between the AFM tip and the surface, revealing variations in friction coefficients across different regions of the sample.

#### 2.2.3 Amplitude and Phase

Amplitude and phase data, both collected in NCM alongside height information, are unique to AFM measurements and can often confuse beginners. In NCM, the amplitude of the oscillating probe is measured, serving a role analogous to deflection in CM. Similar to CM, the amplitude data can be affected by artifacts, such as slope‐related distortions. However, unlike CM, where the tip directly contacts the surface, surface interactions in NCM are mediated by high‐frequency oscillations. Such distinction has spurred the development of techniques that bring the oscillating tip into contact with the sample to probe its mechanical properties, such as force modulation and PeakForce Tapping.^[^ ^17^, ^18^, ^19^ ^]^

Meanwhile, phase data arises from changes in the probe's resonance frequency due to interactions with the surface. These phase variations are sensitive to the strength of attractive and repulsive forces, making phase imaging particularly useful for visualizing surface interactions. Additionally, phase imaging can help differentiate materials on composite surfaces by detecting spatial variations in composition, offering insights into the distribution of different material components. Together, amplitude and phase data enhance the ability to characterize surface properties, including mechanical behavior and compositional differences.

**Figure 2:**
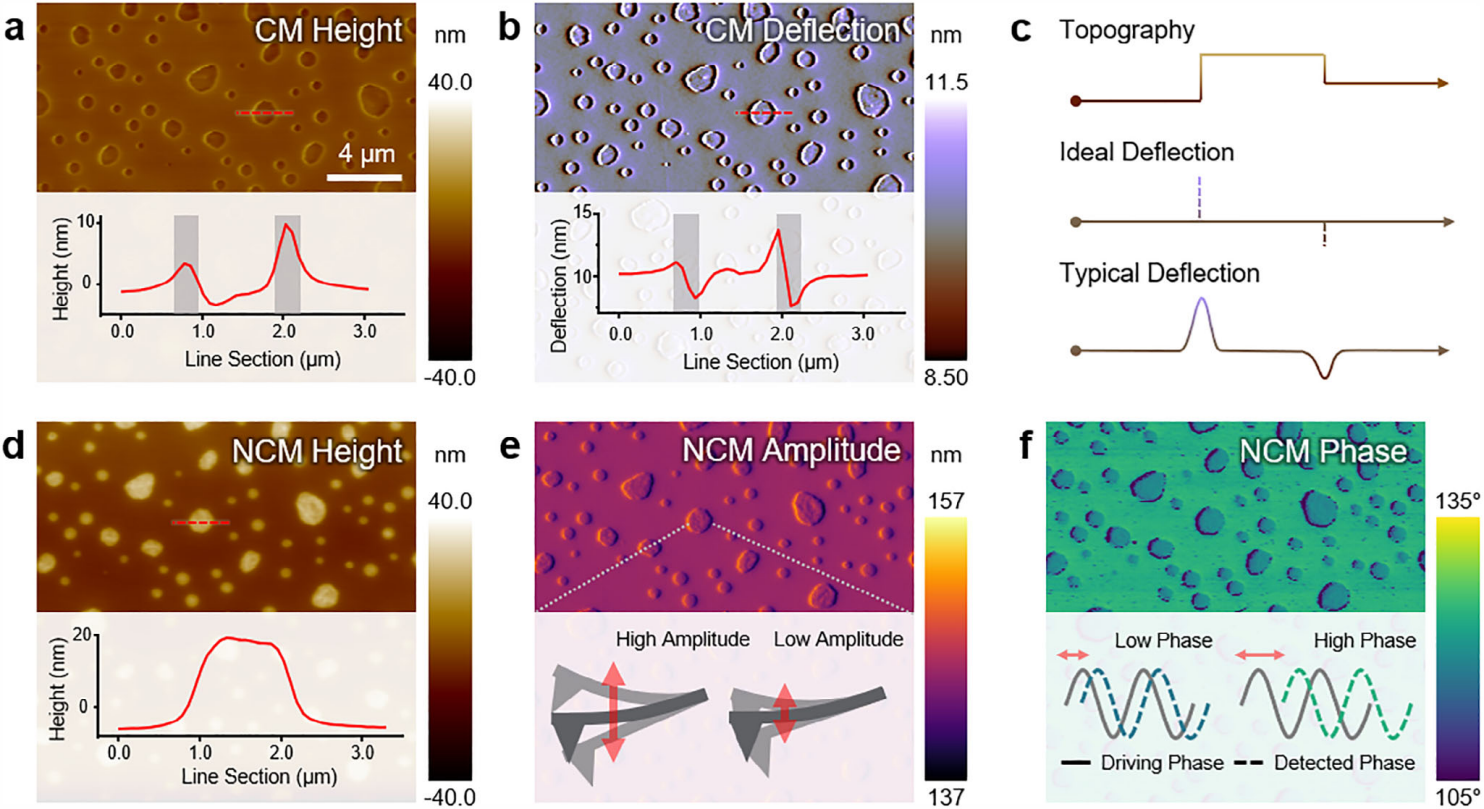
CM and NCM Images of PS‐LDPE. Panels (a–c) correspond to results from CM, while panels (d–f) display results from NCM. In CM height (a), PS and LDPE appear at similar levels, with protrusions visible at the interfaces. These protrusions contribute to error signals, as can be found in CM deflection (b). The schematic in (c) illustrates the ideal zero‐deflection scenario (perfect feedback) expected over a protruded sample feature, contrasted with the actual fluctuation caused by feedback response limitations, highlighted in gray regions of (a) and (b). In comparison, the NCM height (d) reflects differences in the elastic modulus of PS and LDPE, with LDPE regions appearing elevated. Similar to CM deflection, the NCM amplitude (e) shows fluctuations at the interfaces. Meanwhile, it is possible to visualize the spatial variations in stiffness across the sample from the NCM phase (f).

Height, deflection, amplitude, and phase are among the most fundamental parameters obtained from AFM‐based surface analyses. However, because the AFM tip interacts directly with the sample surface, the material properties of the sample can affect the measured data. **Figure** **2** shows the results of analyzing a polystyrene (PS) embedded low‐density polyethylene (LDPE) sample using both CM and NCM. Despite imaging the same sample, the height data differs between the two modes. This discrepancy arises from variations in the mechanical properties, particularly the stiffness, of PS (2 GPa) and LDPE (0.1 GPa).^[^ ^20^ ^]^ Although not the cause of artifacts in this specific example, electrical, magnetic, or even environmental factors can also introduce delusive signals in other types of AFM experiments as well. Therefore, to ensure reliability and accurate analysis, it is crucial to understand the origin of each signal and differentiate true sample features from misleading artifacts.

Beyond its ability to resolve surface features using basic CM and NCM, AFM can be further enhanced by modifying the probe or applying targeted stimuli—such as electrical or magnetic fields—to the tip. This approach enables the investigation of a broad range of material properties, including local conductivity, surface potential, ferroelectric polarization, and other surface characteristics, as discussed below.

#### 2.2.4 Local Conductivity

Among the various physical properties that AFM can measure, local electrical conductivity can be directly accessed by applying a DC voltage through the conducting tip in CM. This technique, referred to as conductive atomic force microscopy (C‐AFM), utilizes the stable tip‐sample junction to measure current flow with nanoscale precision. The continuous nanosized electrical contact ensures excellent spatial resolution, enabling the creation of detailed 2D conductance maps that reveal electrical heterogeneities across the sample surface. This capability makes C‐AFM particularly valuable for investigating local electronic properties in materials ranging from semiconductors to novel electronic devices.

#### 2.2.5 Surface Potential

Just as objects with different electrical potentials experience attractive or repulsive forces, AFM can detect these electrostatic interactions at the nanoscale. KPFM or scanning Kelvin probe microscopy (SKPM) exploits this principle by applying an AC voltage between tip and sample, causing the cantilever to oscillate due to electrostatic forces arising from contact potential differences. By finding the exact DC voltage needed to nullify these oscillations, KPFM maps local surface potential variations, revealing electronic properties like work function distribution and surface charge patterns.

#### 2.2.6 Capacitance

Capacitance‐based AFM techniques probe subsurface electrical properties by measuring local dielectric responses. Scanning capacitance microscopy (SCM) quantifies differential capacitance (∂C/∂V) between a conductive tip and the sample under an applied AC signal with a swept DC bias.^[^ ^21^ ^]^ This method, typically operated in contact mode, is effective for mapping dopant concentration and carrier profiles in semiconductor structures. A related technique, scanning capacitance force microscopy (SCFM), infers capacitive behavior indirectly by detecting electrostatic forces resulting from capacitance gradients (dC/dz), measured via cantilever deflection in lift mode.^[^ ^22^ ^]^ While SCM requires a dedicated capacitance sensor, SCFM can be implemented on standard AFM setups using voltage modulation. Detailed applications of both techniques in semiconductor device characterization are discussed in Section 6.2.

#### 2.2.7 Ferroelectric Polarization and Domain Structure

Ferroelectric materials uniquely respond to electric fields by changing their physical dimensions—a property known as the inverse piezoelectric effect. PFM leverages this behavior by applying an AC voltage through the tip in CM, causing periodic expansion and contraction of the sample surface. High‐frequency AC signals enable clear differentiation between domain response and topographical features, allowing precise mapping of domain walls and local polarization dynamics. This technique has proven crucial for understanding domain switching mechanisms, polarization fatigue processes, and interfacial coupling in complex oxide heterostructures.^[^ ^23^, ^24^, ^25^ ^]^ Moreover, PFM can simultaneously map out‐of‐plane and in‐plane polarization components, providing comprehensive insight into three‐dimensional polarization structures.^[^ ^26^ ^]^

#### 2.2.8 Ion Concentration and Mobility

When ions redistribute within a material, they cause subtle changes in local volume—much like how a crowd of people affects the space they occupy. Electrochemical strain microscopy (ESM) detects these volume changes (Vegard strains) that occur when an AC voltage drives ions to move.^[^ ^27^ ^]^ By correlating the strain response with the applied AC bias frequency, ESM can distinguish between different ionic processes and their activation energies.^[^ ^28^ ^]^ This capability has revolutionized the understanding of ionic conductors, particularly in battery materials where local variations in ion mobility can critically affect device performance.^[^ ^29^ ^]^ The technique can also map electrochemical reactivity at interfaces and grain boundaries, providing insights into degradation mechanisms and ion transport pathways.^[^ ^30^ ^]^

#### 2.2.9 Magnetic Polarization and Domain Structure

Similar to how a compass needle aligns with Earth's magnetic field, a magnetized AFM tip responds to local magnetic fields near a sample's surface. Magnetic force microscopy (MFM) uses this interaction to map magnetic domains and field patterns without making physical contact.^[^ ^31^ ^]^ The technique's ability to operate under various environmental conditions makes it invaluable for studying magnetic recording media, spintronic devices, and novel magnetic materials.^[^ ^32^, ^33^, ^34^ ^]^ By carefully controlling the tip‐sample separation, MFM can achieve resolutions down to tens of nanometers, enabling detailed studies of domain wall dynamics and magnetic switching behavior.^[^ ^35^ ^]^

#### 2.2.10 Charge Distribution and Polarization Mapping

Much like how dust particles can obscure a surface pattern, mobile charges in air or solution can screen underlying charge distributions on surfaces. Charge gradient microscopy (CGM) overcomes this by rapidly scanning a conductive tip across the surface, displacing these screening charges temporarily.^[^ ^36^ ^]^ This dynamic approach enables direct visualization of hidden charge patterns without external bias application, revealing inherent polarization distributions in materials. The technique's high scanning speed minimizes drift and environmental effects, making it particularly effective for studying dynamic charge processes and surface electrochemical phenomena. CGM has proven especially valuable in understanding ferroelectric materials and their domain structures, offering complementary information to traditional PFM measurements.^[^ ^37^, ^38^ ^]^

## 3 Data Acquisition and Experiment Protocols

**Figure 3:**
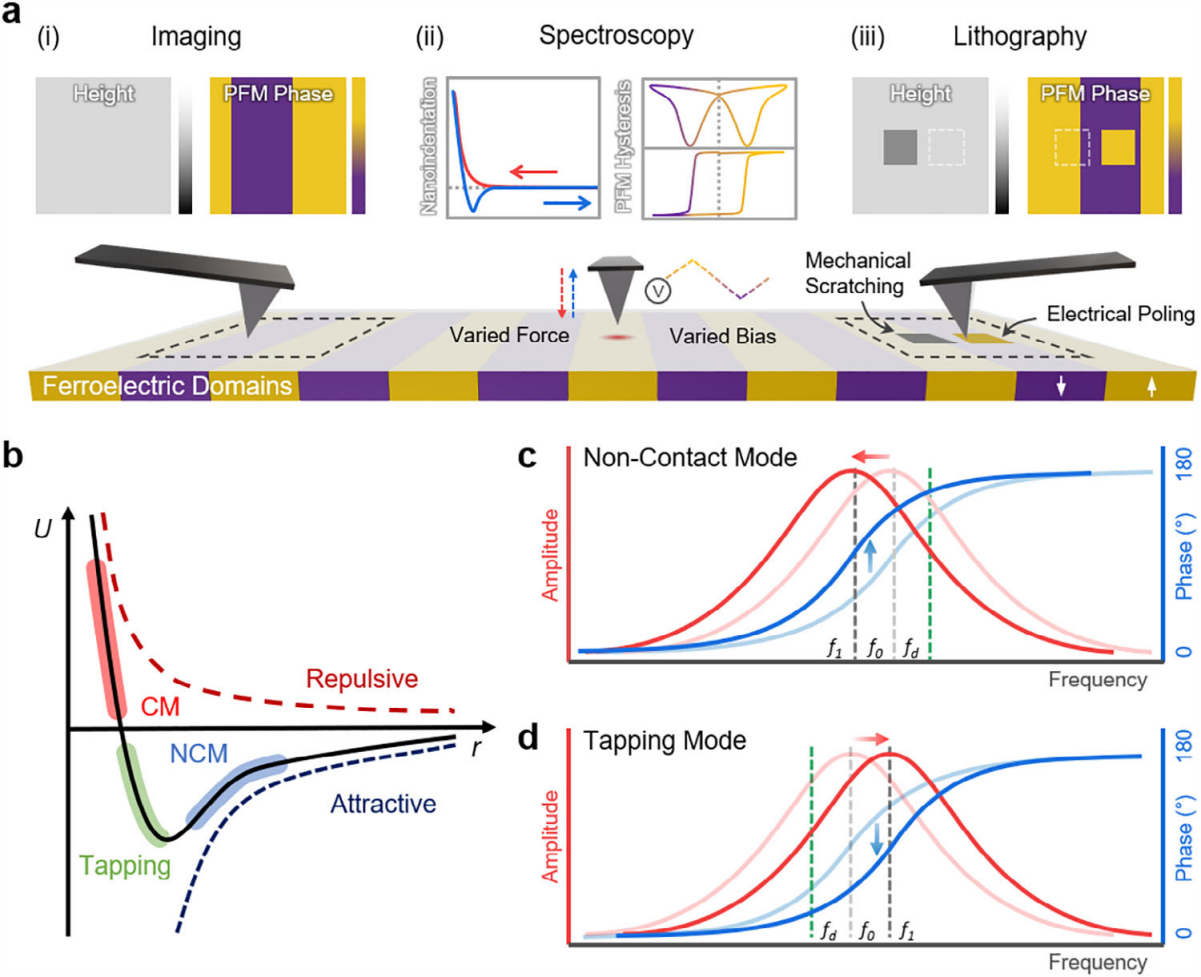
Fundamentals of AFM Surface Characterization. a) An illustration of AFM characterization techniques: i) Imaging, ii) Spectroscopy, and iii) Lithography, with examples demonstrated on a periodically poled lithium niobate (PPLN) sample. Imaging methods include height mapping and PFM phase visualization. Examples of spectroscopy include nano‐indentation measurements (force variation at a fixed point) and PFM hysteresis measurements (bias variation). Lithography examples show mechanical scratching and electrical poling, which respectively create a reduced‐height region in the height image and a reversed polarization region in the PFM phase image. b) AFM tip‐sample distance regimes, mapped onto the total energy profile *U(r)*, illustrate transitions between attractive and repulsive force regimes. These regimes correspond to NCM, tapping, and CM imaging modes. c) In NCM, attractive forces induce a leftward peak amplitude shift and a phase increase, while d) tapping mode shows a rightward peak amplitude shift and a phase decrease under repulsive forces. The resonance frequency of the tip shifts from *f*_0_ (before engagement) to *f*_1_ (after engagement), with *f_d_* representing the drive frequency.

Acquiring high‐quality AFM data demands careful consideration of experimental protocols, appropriate instrument configuration, and a comprehensive understanding of potential measurement artifacts. In this section, we discuss the fundamental aspects of data collection, beginning with an overview of characterization types (imaging, spectroscopy, lithography) (**Figure** **3**a), followed by instrumentation considerations (probe selection, environmental setup, and calibration), and concluding with strategies for identifying and mitigating common measurement artifacts that can compromise data integrity. Researchers can obtain reliable and reproducible measurements to advance their scientific investigations by properly implementing these experimental protocols.

### 3.1 Types of AFM Characterization

#### 3.1.1 Imaging

As described in Section 2.1, AFM imaging modes are typically classified based on the nature and persistence of contact between the tip and the sample surface. Specifically, these modes are distinguished by the tip‐sample surface distance, which affects the dominant interaction forces and the resulting energy profiles. Figure 3b illustrates this relationship, plotting total energy as a function of distance and showing how the dominant interaction forces change. The derivatives of the plot reflect the forces at different distances: repulsive forces dominate to the left of the local energy minimum, while attractive forces prevail to the right. As the tip approaches the sample, it initially enters the attractive force regime utilized in NCM. Moving closer, the tip crosses the local energy minimum toward the repulsive force regime, where the tapping mode primarily operates. Further proximity causes the tip to exert sufficient force to deflect the cantilever, making this region suitable for CM.

##### Contact Mode Imaging

CM represents the most fundamental mode in AFM, where the tip remains in continuous contact with the sample surface under a controlled loading force. This force is determined by three key parameters: the cantilever's spring constant, the setpoint, and the inverse optical lever sensitivity (InvOLS). The loading force can be expressed as Equation (3):

(3) $$\begin{array}{ccc} \mathrm{Loading}\;\mathrm{force}\;\left(N\right) & & =\mathrm{Spring}\;\mathrm{constant}\;\left(N\;m^{-1}\right)\\ & & \times \;\mathrm{Setpoint}\;\left(V\right)\times \;\mathrm{InvOLS}\;\left(m\;V^{-1}\right) \end{array}$$

The spring constant reflects the inherent stiffness of the cantilever, while InvOLS quantifies how the cantilever's bending affects the photodetector. InvOLS, expressed in *m/V*, converts the detector's electrical signal (*V*) into cantilever deflection (*m*). After calibrating the spring constant and InvOLS for each experiment, the setpoint becomes the only tunable parameter, allowing precise control of the loading force as needed.

Choosing the appropriate setpoint requires careful consideration of the sample's material properties. An insufficient loading force may result in unstable tip‐sample contact, leading to poor surface tracking and unreliable measurements. Conversely, excessive force can damage both the sample and the tip, potentially causing cantilever failure due to extreme bending. Therefore, achieving the right balance—ensuring stable contact while avoiding sample damage or tip deformation—is crucial for successful CM measurements. This balance is achieved by accurately calibrating the spring constant and InvOLS before the measurement while maintaining optimal setpoint control throughout the process.

The continuous tip‐sample contact in CM makes it ideal for imaging the topography of flat or relatively rigid surfaces. It also provides a conduction path for electrical measurements, enabling techniques that require stable electrical contacts, such as C‐AFM,^[^ ^39^, ^40^, ^41^ ^]^ PFM,^[^ ^42^, ^43^, ^44^, ^45^ ^]^ and ESM.^[^ ^46^, ^47^ ^]^

##### Non‐Contact Mode Imaging

Imagine a finger hovering just above a surface while sensitive enough to feel the texture without actually touching it. This is how NCM works, using a continuously oscillating cantilever to sense surface features without making direct contact. The cantilever acts like a tiny tuning fork, vibrating with a precisely controlled amplitude at its natural resonance frequency.

As the oscillating AFM tip approaches the sample surface, it enters a region where tip–sample interactions (like van der Waals forces) begin to influence its motion, requiring the system to maintain a specific setpoint to stay within this sensitive interaction regime. In AM‐AFM, this is achieved by holding the oscillation amplitude at a reduced, constant value, since attractive forces cause a slight damping of the cantilever's motion. In contrast, FM‐AFM monitors changes in the cantilever's resonance frequency instead of amplitude. Here, variations in the local force gradient (∂F/∂z) near the surface lead to shifts in the resonance frequency, and the system keeps this frequency shift constant to probe the same interaction regime. In both modes, as the tip scans across areas of differing height or material properties, the system dynamically adjusts the tip's vertical position to preserve the chosen setpoint. But the signal being regulated differs, reflecting the distinct physical quantities each mode is sensitive to.

This delicate interaction yields two key pieces of information. First, height adjustments are needed to maintain constant amplitude or frequency shift and generate a topographical map. Second, the phase difference between the driving oscillation and the cantilever's response provides insights into surface properties—similar to how touching different materials (like metal versus wood) gives distinct tactile feedback. This phase information is susceptible to material properties such as viscosity, adhesion, and elasticity variations.^[^ ^48^, ^49^, ^50^ ^]^

NCM's non‐invasive nature makes it ideal for several specialized applications. For instance, when investigating electrical or magnetic properties (using techniques such as electrostatic force microscopy (EFM),^[^ ^51^ ^]^ KPFM,^[^ ^52^, ^53^ ^]^ or MFM)^[^ ^54^, ^55^ ^]^ maintaining a consistent tip‐sample distance preserves the strength of the applied force fields on the sample surface. This gentle approach is also practical for investigating soft or fragile samples that could be damaged by direct contact, allowing for high‐resolution imaging while preserving sample integrity.

One such technique is EFM, which extends the non‐contact mode to detect long‐range electrostatic interactions. It typically operates in a two‐pass scanning mode: the first pass acquires topography, and the second retraces the same line at a fixed lift height. During the second pass, a voltage is applied between the conductive tip and sample, generating electrostatic forces that modulate the cantilever's oscillation through phase shift or frequency change. These modulations reflect variations in local surface potential or charge distribution. As the tip remains physically separated from the surface, EFM is suitable for probing electrostatic properties in insulating or compositionally heterogeneous samples.

An important extension of non‐contact mode is three‐dimensional atomic force microscopy (3D‐AFM), which enables the quantitative mapping of tip–sample interaction fields with high spatial resolution in all three dimensions.^[^ ^56^ ^]^ While conventional NCM provides topographic images based on constant frequency shift contours, 3D‐AFM reconstructs full force and energy landscapes by acquiring frequency shift data across a dense grid of lateral and vertical positions. In this method, either a sequence of frequency shift curves (Δ*f*(*z*)) is recorded at each lateral pixel or a stack of constant‐height Δ*f*(*x*,*y*) images is acquired at varying tip–sample distances. These datasets are then mathematically converted to site‐specific maps of interaction forces, potential energy, and energy dissipation. The technique has been successfully applied to materials such as graphite and molecular adsorbates, revealing spatially resolved force fields and lateral force components that are inaccessible to conventional topographic imaging. 3D‐AFM thus expands the capabilities of NCM by providing detailed physical insight into surface interactions, with implications for chemical imaging, catalysis, and nanotribology.

##### Multifrequency AFM

Amplitude–Frequency Modulation (AM‐FM), also referred to as bimodal AFM, enables simultaneous acquisition of surface morphology and nanomechanical properties.^[^ ^57^, ^58^ ^]^ This mode excites the cantilever at two distinct resonance frequencies: the first for topographical feedback and the second for detecting local stiffness variations. The amplitude and frequency shift of the second mode are analyzed to extract quantitative mechanical parameters such as elastic modulus and energy dissipation. Since each eigenmode responds independently to different force components, AM‐FM provides enhanced contrast in heterogeneous materials.

##### High‐Speed AFM

High‐speed atomic force microscopy (HS‐AFM) extends conventional AFM capabilities by enabling real‐time imaging at sub‐second frame rates. This advancement is achieved through the integration of fast scanning piezo stages, high‐bandwidth feedback control, and the use of ultra‐lightweight cantilevers with reduced spring constants. To minimize tip‐sample interaction forces at high speeds, HS‐AFM often employs off‐resonance tapping or photothermal actuation modes such as photothermal off‐resonance tapping (PORT).^[^ ^59^ ^]^ These allow stable operation in soft and dynamic biological systems while maintaining nanometer‐scale spatial resolution. In parallel, image acquisition systems and automated processing algorithms, including AI‐based tools, have been developed to handle high‐volume data streams. The combination of hardware miniaturization, precision control systems, and advanced signal processing has made HS‐AFM a critical technique for observing nanoscale structural dynamics, particularly in biomolecular and soft‐matter systems (See Section 6.5).

#### 3.1.2 Spectroscopy

While AFM imaging provides a comprehensive surface map, AFM spectroscopy reveals detailed material properties at specific points. Think of it as the difference between looking at a photograph of a surface versus poking it with a highly sensitive probe where each interaction can reveal distinct characteristics of that exact spot. These point‐contact analyses typically include electrical and mechanical measurements, where an external bias or mechanical force is applied through the AFM probe at designated locations. Statistical analysis can be conducted by measuring multiple points across the surface. Although common types of AFM spectroscopy involve current–voltage (*I*–*V*) spectroscopy for electrical measurements and nanoindentation for mechanical properties, additional measurements are also possible, such as PFM hysteresis for studying dielectric materials, among other specialized types of analyses. It is helpful to recognize that, despite their conceptual similarities, these techniques are referred to by different names across AFM platforms.

In *I*–*V* spectroscopy,^[^ ^60^, ^61^ ^]^ the AFM tip acts as a nanoscale electrical probe. Once precise electrical contact is established between the tip and the sample, the system applies a controlled voltage pattern and measures the resulting current flow. This process, analogous to testing an electronic component but at nanoscale dimensions, generates *I*–*V* curves that unveil local electrical properties. These measurements can reveal critical information about conductivity mechanisms, electronic barriers, and charge transport behaviors at specific locations on the sample.

Nanoindentation,^[^ ^62^ ^]^ also referred as PinPoint mode, represents AFM's ability to perform extremely localized “push‐and‐pull” tests. This technique generates force‐distance curves that encode various mechanical properties by precisely controlling how the tip presses into and withdraws from the surface. As pressing fingers against different materials tell about their hardness and elasticity, these measurements reveal local elastic modulus, adhesion forces, and material deformation behaviors. The technique's precision allows for detailed mapping of mechanical properties across a sample by performing measurements in a grid pattern, building a comprehensive understanding of spatial variation in material properties.

Alternatively, vibration‐assisted modes like Pulsed Force Mode,^[^ ^63^ ^]^ Sub‐resonance Tapping Mode,^[^ ^64^ ^]^ and PeakForce Tapping^[^ ^65^ ^]^ enable mechanical characterization by analyzing dynamic tip‐sample interactions. These techniques are widely employed due to their compatibility with high‐speed scanning and minimal lateral forces, in contrast to indentation‐based methods like nanoindentation and PinPoint mode.

#### 3.1.3 Lithography

The AFM tip's precision control enables it to function not only as an imaging tool but also as a nanoscale “pen” for surface modification. The AFM tip can directly modify and pattern surfaces through mechanical, thermal, and electrical approaches. This scanning probe lithography (SPL) capability transforms the AFM into a sensitive platform for nanoscale engineering, enabling controlled surface modifications with nanometer precision.^[^ ^66^ ^]^

Mechanical lithography^[^ ^67^, ^68^ ^]^ is microscopic sculpting, and the AFM tip, acting like a precisely controlled chisel, can physically shape the surface through controlled force application. By carefully adjusting the applied force, researchers can create intricate patterns or selectively modify specific regions based on material hardness. The resolution of these modifications, primarily determined by the tip's geometry, size, and force control, can reach dimensions of just a few nanometers.

Thermal lithography (t‐SPL)^[^ ^69^, ^70^ ^]^ introduces a new dimension using a heated tip, like a miniature soldering iron, to induce local material changes. The controlled application of heat can trigger various material responses, from polymer restructuring to time‐dependent phase transitions, enabling precise pattern formation. This technique proves valuable for heat‐sensitive materials where traditional mechanical approaches might be too destructive.

Electrical lithography^[^ ^71^ ^]^ harnesses electric fields between the tip and sample to manipulate materials at the nanoscale. It includes current‐induced manipulation and electric field‐induced switching in ferroelectric materials. Controlled electric fields can induce local modifications in materials, particularly useful in ferroelectric materials and semiconductor devices. This approach creates patterns and provides insights into how materials respond to electrical stimuli at the nanoscale.

The latest advancement in AFM lithography extends into dimensions beneath the surface through tomographic techniques (3D tomography).^[^ ^72^ ^]^ AFM can now construct 3D visualizations of internal structures by systematically removing material layers while mapping properties. This technique involves controlled material removal and layer‐by‐layer property mapping through sequential scanning with calibrated loading forces. The resulting data is reconstructed into comprehensive 3D visualizations, revealing previously inaccessible features such as subsurface structures, defect networks, and spatial variations in material properties.

### 3.2 Instrumentation and Setup

Given AFM's diverse measurement techniques and capabilities, users must select the appropriate instrumentation and setup for each measurement. The AFM must be configured according to the material's properties or the particular characteristic to be measured. Generally, more advanced AFM modes require a more complex experimental setup. This section offers a user‐friendly guide to AFM setup, from selecting the proper probe to configuring environmental settings and performing calibrations.

#### 3.2.1 Probe Selection

The first step in setting up an AFM experiment is selecting an appropriate tip and cantilever combination that aligns with the specific experimental requirements. Several key parameters must be considered when choosing an AFM probe, including cantilever stiffness, tip materials (coating materials for electrical conductivity), and tip geometry.

##### Cantilever Stiffness

The stiffness of the cantilever is fundamental in determining measurement quality through its influence on tip‐sample interaction dynamics. A commonly referenced spring constant to differentiate between flexible and stiff cantilevers is 2.8 N m^−1^, though this value serves only as a general guideline. The selection of cantilever parameters should primarily be based on compatibility with the sample's surface characteristics.

Low spring constant cantilevers (<2.8 N m^−1^) provide enhanced force sensitivity through larger deflections per unit force, making them ideal for soft samples where minimal force application is crucial. However, this increased sensitivity comes with a trade‐off; these flexible cantilevers are more susceptible to environmental noise and surface adhesion forces, which can compromise measurement stability.

In contrast, high spring constant cantilevers (>2.8 N m^−1^) offer distinct advantages in measurement stability. Their increased stiffness enables them to maintain consistent tip‐sample contact forces, improving image quality, particularly in CM measurements. This enhanced force control stems from the cantilever's ability to overcome local interaction energy variations in contact with the surface. The higher spring constant also reduces the influence of environmental vibrations on measurement quality,^[^ ^73^ ^]^ as the cantilever requires greater force to induce significant deflection.

On the other hand, in NCM, the quality (Q) factor is critical in selecting appropriate cantilevers, reflecting the system's sensitivity to tip‐sample interactions. Cantilevers with higher Q‐factors, often associated with stiffer materials and operation in low‐damping environments, offer improved resolution and force sensitivity. Excessively high Q‐factors, however, may compromise feedback stability.

The choice of cantilever stiffness thus becomes a critical experimental parameter that must be optimized based on both sample properties and measurement objectives. For instance, while soft cantilevers excel at mapping polymer films, liquid interfaces, and delicate biological samples where minimal perturbation is essential, stiff cantilevers are preferred for high‐resolution imaging of rigid surfaces where consistent tip‐sample contact is paramount for data quality.

##### Tip Materials and Geometry

The AFM tip is the primary interface between the instrument and sample, where its material composition and geometry fundamentally determine measurement capabilities and quality. At its core, the tip must be engineered to optimize specific tip‐sample interactions for different measurement modes. Conductive coatings like platinum (Pt), iridium (Ir), or gold (Au) enable electrical measurements using techniques such as KPFM, PFM, and C‐AFM, while advanced surface modifications can enhance measurement stability and precision.

Modern tip engineering extends far beyond basic metal coatings. Surface functionalization through polymer coatings, self‐assembled monolayers, or specific chemical treatments can precisely tune tip‐sample interactions.^[^ ^74^ ^]^ These modifications can control surface energy, enhance chemical specificity, or modify wettability, opening new possibilities for specialized measurements. For instance, hydrophobic coatings can improve performance in liquid environments, while functionalized polymer layers can enable selective molecular recognition in biological applications.^[^ ^75^, ^76^ ^]^

Tip geometry plays an equally important role in measurement quality. The tip's shape, diameter, and aspect ratio directly influence its ability to track surface features accurately. While sharp tips with small radii (a few nanometers) maximize spatial resolution, they may struggle with deep or steep features or from electric field‐induced cleavage. Conversely, tips with higher aspect ratios better track complex topographies but may sacrifice ultimate resolution.

In addition to imaging, the tip's geometry also significantly affects quantitative measurements in techniques such as nanoindentation.^[^ ^77^ ^]^ Precise knowledge of the contact mechanics, including shape and associated contact area, is required for accurately determining mechanical quantities. Common tip geometries include spherical, conical, and square pyramidal shapes, leading to distinct contact areas in the calculation based on contact mechanics theories.^[^ ^78^ ^]^ Therefore, accurate information about the tip geometry should primarily be recognized either from provided specifications or scanning electron microscopy (SEM) imaging.

This complexity in tip design reflects a fundamental truth in AFM measurements, and the probe must be carefully matched to both the sample characteristics and measurement objectives. While advanced tip modifications can significantly enhance measurement capabilities, they also increase costs, making careful consideration of requirements essential for optimal experimental design.

#### 3.2.2 Environmental Setup

Creating and maintaining an optimal environmental setup is as crucial as the interaction between the AFM tip and the sample to achieve successful experimental outcomes. Key factors include the cantilever holder and environmental controls designed to create and maintain specific experimental conditions.

##### Cantilever Holder

The cantilever holder is the crucial connecting interface between the probe and the AFM system. The holder must provide the perfect cantilever alignment, stable electrical connections, and sometimes specialized functionalities for specific measurement modes. Basic holders must achieve precise mechanical coupling, as even microscopic misalignments can introduce significant measurement artifacts. In AFM, such instability manifests as asymmetric scanning patterns, signal drift, or unreliable force control. The mounting screws must therefore be tightened with exact torque; too loose and the cantilever moves, and too tight and one risks damaging the delicate components.

Advanced measurements demand increasingly versatile holder designs. C‐AFM requires holders with integrated electrical pathways to carry stable signals while minimizing noise less than the fA scale. Electrochemical studies need holders resistant to chemical reactions such as corrosive environments, often incorporating specialized coatings or materials. Some measurements might even require temperature control or optical access, further complicating holder design. Each additional requirement introduces new potential failure points that must be carefully managed.

##### Equipment for Environmental Setup

Environmental control in AFM encompasses much more than simple temperature and humidity regulation. It creates the conditions necessary for reliable measurements, particularly advanced characterization techniques. This control becomes increasingly complex as measurement requirements become more demanding. Consider electrical measurements such as C‐AFM or KPFM: atmospheric moisture can create unwanted conductive pathways or interfere with electric fields,^[^ ^73^ ^]^ compromising measurement accuracy. The solution involves creating controlled atmospheres using inert gases. Every connection point becomes a potential leak source, each electrical feedthrough must maintain signal integrity while ensuring environmental isolation, and temperature gradients must be carefully managed to prevent drift.

The complexity compounds when combining multiple requirements. For example, electrochemical measurements might need environmental isolation and specific atmospheric conditions while maintaining electrical connections and chemical resistance. Even simple humidity control requirements can become challenging when maintaining stable conditions over long measurement periods. Success in these setups requires understanding how each component functions and how they interact. A perfectly sealed chamber means nothing if it introduces mechanical vibrations, just as excellent electrical connections are useless if they compromise environmental control. This interdependence of requirements explains why seemingly straightforward measurements can require surprisingly complex experimental setups.

#### 3.2.3 Measurement Calibrations

Each AFM probe has unique properties, such as cantilever spring constants, InvOLS values, and other characteristics, making precise calibration essential for accurate imaging. Calibration ensures controlled tip‐sample interaction, reducing the risk of sample damage during imaging. Calibration also prevents data misinterpretation, with specific calibration methods for each mode, particularly for offset adjustments.

##### Calibrations of the Cantilever

Due to the inherent variability in manufacturing processes, tips from the same cassette cannot be perfectly identical. Therefore, tip calibration is mandatory to achieve accurate imaging. In CM, the cantilever's spring constant is calculated through thermal tuning. The cantilever's thermal fluctuations are measured over time and converted to mechanical responses, representing a frequency spectrum. The spring constant is then determined by fitting the resonance peak. InvOLS tuning follows the thermal tuning process, with selecting a sufficiently rigid sample being crucial for accurate calibration. This ensures the sample remains intact as the probe approaches the surface, allowing the cantilever to bend without digging into the sample. To obtain reliable spring constant and InvOLS values, it is recommended that the calibration process be performed at least three times to achieve convergence. These values enable precise loading force calculations with an appropriate setpoint.

In AM‐AFM, the tip resonance frequency is determined through tip‐tuning, ensuring precise engagement with the sample. This calibration process verifies the approach's accuracy and emphasizes the importance of calibration in NCM imaging. As illustrated in Figure 3c,d, changes in amplitude and phase occur in opposite directions in NCM and tapping mode, depending on whether attractive or repulsive forces dominate. In NCM, operating under attractive forces, the peak amplitude shifts left, and the phase increases as the tip approaches the sample (Figure 3c). In tapping mode, where more repulsive forces dominate, the peak amplitude shifts right, and the phase decreases as the sample moves closer (Figure 3d). For accurate measurements, the drive frequency should be set slightly to the right (+%) of the center peak in NCM and slightly to the left (–%) in tapping mode. Although NCM and tapping modes share similarities, the mode choice often depends on the sample's specific properties. Proper tuning through calibration is crucial to ensure accurate measurements in the desired mode.^[^ ^79^, ^80^ ^]^

In FM‐AFM, calibration similarly begins with tip tuning to identify the precise resonance frequency of the cantilever under free oscillation conditions. Unlike AM‐AFM, FM‐AFM maintains continuous oscillation exactly at the resonance frequency, which shifts in response to changes in the tip–sample force gradient. This frequency shift serves as the primary signal for imaging and feedback control. Accurate detection of this shift requires a stable phase‐locked loop or frequency demodulator, and the cantilever's Q‐factor and oscillation amplitude must be carefully optimized during calibration to ensure sensitivity and resolution, particularly in low‐Q environments such as liquid.^[^ ^81^, ^82^, ^83^ ^]^

##### Calibrations for Environmental Changes

So far, calibrations have been discussed in the context of measurements performed under standard atmospheric conditions. When environmental conditions are controlled, it is necessary to verify that measurements are accurate within the controlled environment. Depending on the sample's nature, achieving precise measurements may require isolating the sample from the external atmosphere. Introducing inert gas during the experiment may also be necessary. However, the introduction of gas can affect AFM tip movement due to changes in hydraulic pressure. Thus, it is advisable to conduct preliminary measurements with test samples to determine the optimal conditions for environmental control (e.g., type of gas, flow rate) before proceeding with actual sample measurements.

Similarly, when measurements are performed in liquid, the system is fully immersed, meaning that the space between the back of the cantilever and the PSPD—typically filled with air—would be filled with liquid. The refractive index difference between air and the liquid medium can cause shifts in the light's position, requiring adjustments. Before the primary measurements, the reflected light from the cantilever must be corrected to ensure proper alignment with the PSPD origin. Since InvOLS values are also influenced by refraction, values obtained from calibration under atmospheric conditions should not be used for liquid measurements. As with CM calibration, the process should be repeated with a rigid sample, but the InvOLS value must be recalibrated in the liquid medium.

##### Calibrations for Advanced Modes

In advanced measurement modes, the calibration of additional parameters is often required. These processes ensure the reliability and accuracy of the AFM tip using materials with well‐known physical properties. For instance, in KPFM, the tip's work function is calibrated by measuring a reference material with a known work function, such as gold or highly oriented pyrolytic graphite (HOPG). Similarly, for nanoindentation, the tip is initially calibrated using materials with established elastic moduli, such as PS‐LDPE. Such steps ensure that key parameters, such as tip geometry and fitting models, are properly selected, ensuring stable and reproducible measurements across experiments.

### 3.3 Addressing Crosstalk and Artifacts

While AFM drives advances in materials science, it also presents various challenges that must be carefully managed to ensure accurate measurements. The key to maintaining the integrity of AFM results is recognizing and addressing potential artifact sources. AFM probes are influenced by a complex interaction of physical, chemical, and mechanical factors when interacting with surfaces at the nanoscale. As a result, AFM‐based analysis can sometimes yield artifacts that do not accurately reflect the intended signals, distorting data and leading to misinterpretations. In some cases, these artifacts can appear more prominent than the targeted signal itself, causing significant image distortion and misreading of material properties.^[^ ^27^, ^84^, ^85^ ^]^

**Figure 4:**
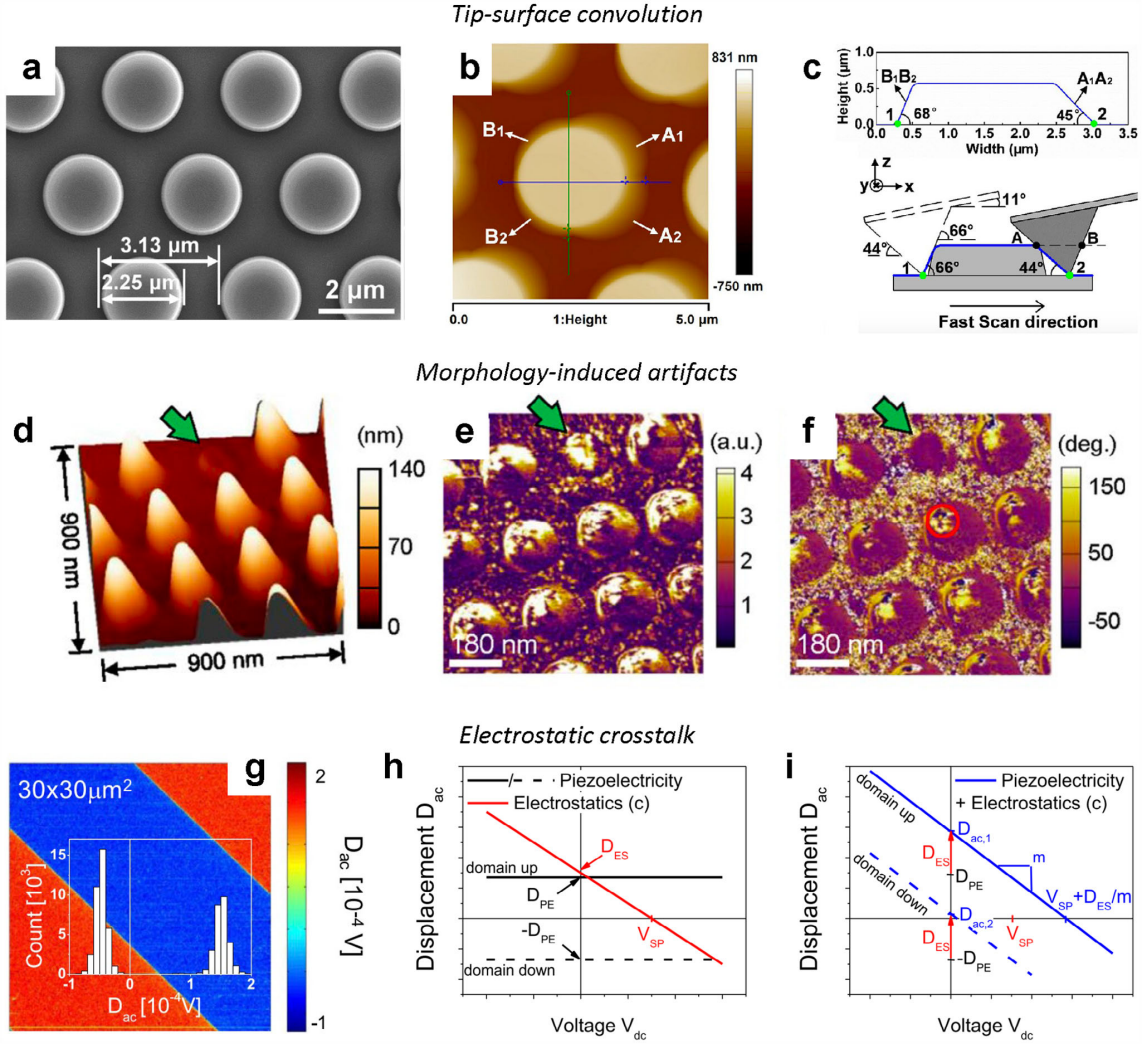
Crosstalk and Artifacts in AFM Characterizations. Illustration of tip‐surface convolution (a–c), morphology‐induced artifacts (d–f), and electrostatic crosstalk (g–i). a) SEM top‐view image and b) AFM topography image of a patterned sapphire substrate with cylinders. c) The cross‐section profile along the green line in (b) with a schematic illustrating the tip‐cylinder interaction, highlights convolution effects on both sides of the cylinder. Reproduced with permission.^[^ ^86^ ^]^ Copyright 2017 Elsevier. d) 3D AFM topography image of BTO/STO heterostructures, with single‐frequency PFM amplitude (e) and phase (f) images. The green arrows indicate a region where a pillar is missing in the scanned region. Reproduced with permission.^[^ ^91^ ^]^ Copyright 2016 AIP Publishing. g) PFM image of domains on a PPLN crystal, with h) isolated piezoelectric signals and i) combined piezoelectric and electrostatic responses, emphasizing the effects of electrostatic crosstalk. Reproduced with permission.^[^ ^95^ ^]^ Copyright 2016 IOP Publishing.

Topography distortions due to tip‐surface convolution are major artifacts in AFM imaging, particularly affecting the accuracy of surface morphology interpretation.^[^ ^86^, ^87^, ^88^ ^]^ For example, as illustrated in **Figure** **4**a, an SEM image of micron‐sized cylindrical patterns shows their true shape. However, when the same sample is imaged using AFM (Figure 4b), the resulting topography image exhibits broadened, arc‐shaped zones around the cylinders.^[^ ^86^ ^]^ This distortion occurs because the AFM tip's geometry cannot fully conform to the surface contours, as shown in Figure 4c. Comparing trace and retrace images or re‐scanning the sample upon rotation is recommended to verify such artifacts. Optimizing scan parameters, such as setpoint, integral gain, and scan rate, can help improve the tracking of surface features. Using a high‐aspect ratio or tilted sharp‐tip probes can reduce convolution effects, resulting in more accurate topographic images. Additionally, sample drift in CM can introduce artifacts, especially in samples with deposited particles or piled structures. NCM imaging might yield more accurate results than CM in such cases, provided that the setpoint and scan rate are carefully adjusted.

The sample's surface morphologies in AFM characterization can also distort acquired signal images and data. These artifacts occur due to mechanical coupling between the tip and surface features, which induces frequency shifts during scanning and can lead to misinterpretation of the target response. Such morphological artifacts can occur in both CM‐based or NCM‐based modes, including friction force microscopy (FFM),^[^ ^89^ ^]^ PFM,^[^ ^90^, ^91^ ^]^ and KPFM.^[^ ^92^ ^]^ In PFM, for example, the phase and amplitude signals should ideally align spatially to distinguish piezoelectric response regions from background noise. However, in Figure 4d–f, it is shown that the amplitude and phase of a pillar structure coated with a ferroelectric film show contrast over partial halves of the pillars, suggesting artifacts that obscure the true piezoelectric response.^[^ ^91^ ^]^ Even on a broken pillar where non‐piezoelectric silicon is exposed, the amplitude signal still appears, indicating artifact‐induced distortion. Minimizing the influence of surface features, reducing the scan rate, or employing cantilevers with high spring constants can help mitigate these artifacts.

Another common artifact is electrostatic crosstalk, which can distort the accurate measurement of electric properties in techniques such as PFM, ESM, or other electrical spectroscopy‐based measurements.^[^ ^93^, ^94^, ^95^ ^]^ Electrostatic crosstalk arises from Coulomb forces between the AFM tip and the sample surface due to capacitive interactions and surface potential differences. These forces can artificially enhance signal amplitude when a continuous DC voltage is applied during on‐field measurements. In PFM, for example, electrostatic contributions can overshadow accurate piezoelectric responses. This aspect is shown in Figure 4g–i, where the combined piezoelectric and electrostatic displacements result in an asymmetric cantilever response, as seen in Figure 4g.^[^ ^95^ ^]^ Techniques such as pulsed DC modes, using high spring constant cantilevers or incorporating surface electrodes can help reduce these artifacts and improve the accuracy of PFM measurements.

On the other hand, artifacts caused by thermal drift in AFM imaging arise from gradual mechanical displacement due to temperature fluctuations, which cause time‐dependent positional shifts between the probe and the sample. This drift primarily affects the slow scan axis, leading to systematic image distortion, particularly over long‐duration scans. To minimize this effect, an offline correction method that employs cross‐diagonal scanning, template matching, and adaptive filtering to quantify and compensate for thermal drift without requiring hardware modifications is reported.^[^ ^96^ ^]^

Noise is another significant AFM imaging issue affecting measurement accuracy and data reliability. Electronic interferences, often caused by feedback circuit instability or noise within electronic components in the AFM system or even from the heavily connected electrical ground, can introduce image distortions. Environmental vibrations, such as those from nearby machinery or traffic, can further aggravate noise artifacts. These vibrations span a range of frequencies, with mechanical vibrations typically occurring at lower frequencies. To mitigate this, vibration isolation systems or conducting measurements during quieter periods, such as nighttime, are recommended. Analyzing the frequency spectrum of periodic noise features in the images can help identify specific sources of vibration and guide effective noise reduction strategies.

## 4 Data Analysis and Interpretation

### 4.1 Image Processing and Software Tools

While AFM is undoubtedly a powerful method for imaging surfaces at the nanoscale, raw AFM data often contain artifacts such as noise, tilted surfaces, and tip degradation that can obscure accurate analysis or hinder quantitative measurements. Therefore, image processing using specialized software tools is required to address these challenges, for researchers to remove artifacts, enhance data clarity, and perform detailed analyses. However, it must also be noted that image processing must be applied carefully, as it can inadvertently hide or dilute important features of an image. Moreover, over‐processing can distort data or even generate artificial features, leading to misleading conclusions. To avoid these pitfalls, it is important to understand the exact mechanisms of each processing technique and to know when and how it should be applied.

#### 4.1.1 Flattening

AFM does not capture an entire image at once; instead, the AFM probe collects data as it scans the desired region line by line. These horizontal lines are referred to as scan lines, and the individual pixels within each line are defined as scan points. Typically, the data within a single scan line appears continuous and smooth. However, a larger time gap exists between successive scan lines than between the scan points within a line. This temporal difference often leads to artifacts appearing primarily between scan lines, such as height variations caused by thermal drift or sudden changes in the probe's behavior, such as tip breakage or debris accumulation. When scan line artifacts appear in AFM data, careful consideration of processing methods is essential to maintain data integrity. While simple interpolation between adjacent scan lines might seem convenient for apparent improvements, such approaches can potentially obscure or misrepresent real surface features. Instead, flattening techniques that consider the entire image context offer more robust solutions by appropriately adjusting height data across the scan while preserving the original surface information. This method ensures uniform imaging while maintaining the scientific validity of the measurements.

Flattening is performed by minimizing the differences between scan lines as much as possible. In the most basic form, 0^th^ order flattening, the offset of each scanline is adjusted so that the average value of all lines becomes equal. Beyond this, higher‐order artifacts such as slope (1^st^ order) and bow (2^nd^ order) can be removed, referred to as 1^st^ order and 2^nd^ order flattening. Through these processes, it is possible to eliminate vertical artifacts and correct the overall tilt of the surface.

**Figure 5:**
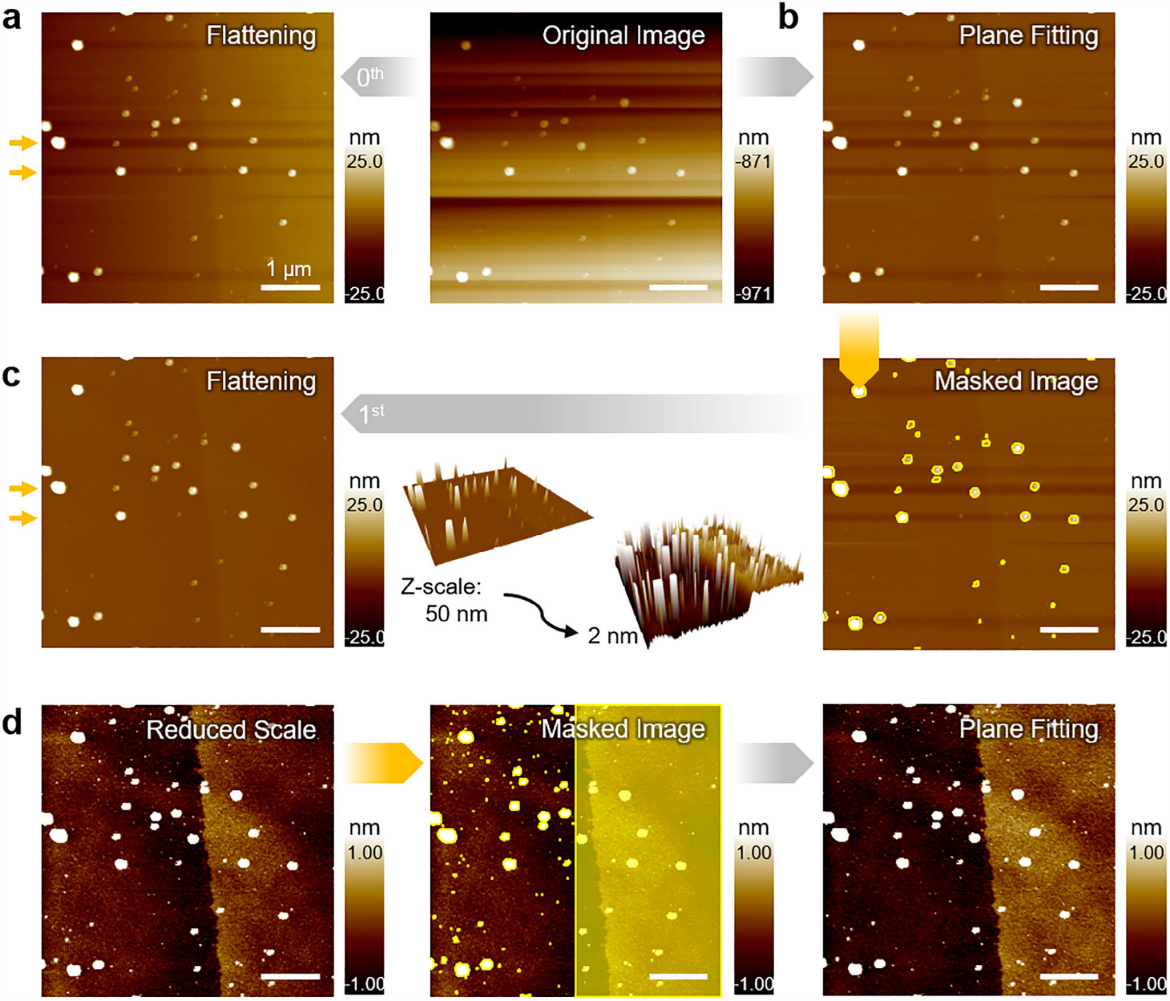
Image Processing Applied to a Topography Image of an Amorphous Carbon Layer. a) A plain 0^th^ order flattening applied to the original image fails to fully correct distortions caused by scattered particles on the surface (marked by yellow arrows). b) A 1^st^ order plane fit along the x‐axis removes the x‐axis slope and still leaves distortions from the particles. c) Masking the particles (outlined in yellow) before flattening eliminates linear artifacts caused by the particles, revealing the stepped surface, which becomes apparent in a 3D view with a modified z‐axis scale. d) Further masking of the upper step, along with the particles, and applying a plane fit significantly reduces the slope artifact, effectively removing all distortions through appropriate masking and plane fitting techniques.

One important consideration during the flattening process is that if the surface is not uniform and contains obvious protrusions, those features can affect the scan line and distort the entire image. For this reason, flattening should either be applied to uniform surfaces where such distortions can be ignored or restricted to substrate regions, excluding the features. **Figure** **5**a illustrates how arbitrary features on a flat surface can distort the image upon a flattening process.

#### 4.1.2 Plane Fitting

Plane fitting is another helpful image correction method when the surface is known to be flat but appears tilted in the image due to thermal drift or sample mounting issues. By fitting and subtracting a plane from the entire image, plane fitting flattens the surface, providing a consistent zero‐height reference for topographical measurements. Unlike flattening, which adjusts line by line, plane fitting operates on the entire image simultaneously, making it more tolerant of large particles or other features that might require masking for flattening.

Plane fitting does not distort the overall shape of the image, so if there are no severe distortions between scan lines, high‐quality images can be achieved using plane fitting alone. However, unlike flattening, which can unintentionally distort the image due to microscale features, macro‐scale features can affect plane fitting. For instance, if the surface is genuinely tilted or if one side is significantly higher, plane fitting may interpret these features as artifacts and distort the image accordingly. Figure 5b shows the result of applying the plane fitting process on a stepped surface.

#### 4.1.3 Filtering

Filtering involves applying modifications to AFM data to remove specific problems, such as noise or unclear edges. While flattening and plane fitting act as effective filters for specific issues, additional filtering algorithms are available for more general applications. Many of these algorithms are adapted from photography and are particularly useful for improving edge visibility. However, it is essential to avoid using filtered images for quantitative measurements, as the modifications may alter the underlying data.

#### 4.1.4 Fast Fourier Transform (FFT)

FFT filtering is a powerful technique for analyzing and removing periodic noise from AFM images. By performing FFT, the frequency components of the image are identified, allowing users to mask and reduce unwanted frequencies. The processed data is then restored using inverse FFT, resulting in clearer images with the periodic noise removed.

#### 4.1.5 Visualization Aids

AFM images are typically rendered in 2D using grayscale or an arbitrary colormap to represent the height at each scan point. However, AFM can acquire multiple data channels on the same surface—some of which may correlate with topography. To assess these correlations, one ordinarily compares the topography image with the property map. By displaying the topography in 3D and projecting the property map onto the surface via a colormap, the spatial relationship between height variations and material properties becomes more intuitively apparent. Additionally, manipulating illumination and shadow effects to accentuate relief, or overlaying contour lines to denote exact height intervals, delivers both qualitative and quantitative clarity, making AFM data more accessible for precise analysis and presentation. In this way, 3D visualization serves as a powerful means to exploit AFM's quantitative imaging capabilities.

When dealing with these image processing techniques, it is important to remember that genuine features of the data can sometimes be mistaken for artifacts. This issue is especially pronounced during flattening, where the per‐scan‐line approach can introduce linear distortions. Filtering can also alter the size of small‐scale features, and FFT might misinterpret recurring nanopatterns as noise. To minimize such risks, analyzing the same region multiple times is recommended to distinguish genuine features from potential artifacts. During this process, adjusting parameters such as scan direction or setpoint can typically either diminish or emphasize artifacts, thereby aiding in differentiating them from the true features.

Handling 3D image data introduces an additional layer of complexity. The representation of z‐axis data—or the choice of scale—can significantly influence how features are perceived, with certain elements either being overlooked or exaggerated. As Figure 5c illustrates, modifying the image scale can shift the emphasis between particles dispersed on the surface and the overall topographical variations. An extreme choice of scale can fundamentally alter the interpretation of results. To mitigate such issues, visualizing the image in 3D can provide a more accurate understanding and help avoid misinterpretation. The key takeaway is that, when conducting AFM analysis and image processing, it is essential to clearly define your objectives and carefully select appropriate scales and techniques to meet them (Figure 5d).

### 4.2 Statistical Methods

A frequently cited limitation of AFM is its restricted field of view, which can make it challenging to capture the overall characteristics of a material. This issue becomes more pronounced when AFM images are treated as isolated visual snapshots, limiting their ability to provide comprehensive insights. However, by treating each pixel as a data point, a single image can serve as a rich source of quantitative information. By using this vast dataset, statistical analysis enables various methods to be applied, tailored to the image type and the specific goals of the analysis.

#### 4.2.1 Line Profiling

**Figure 6:**
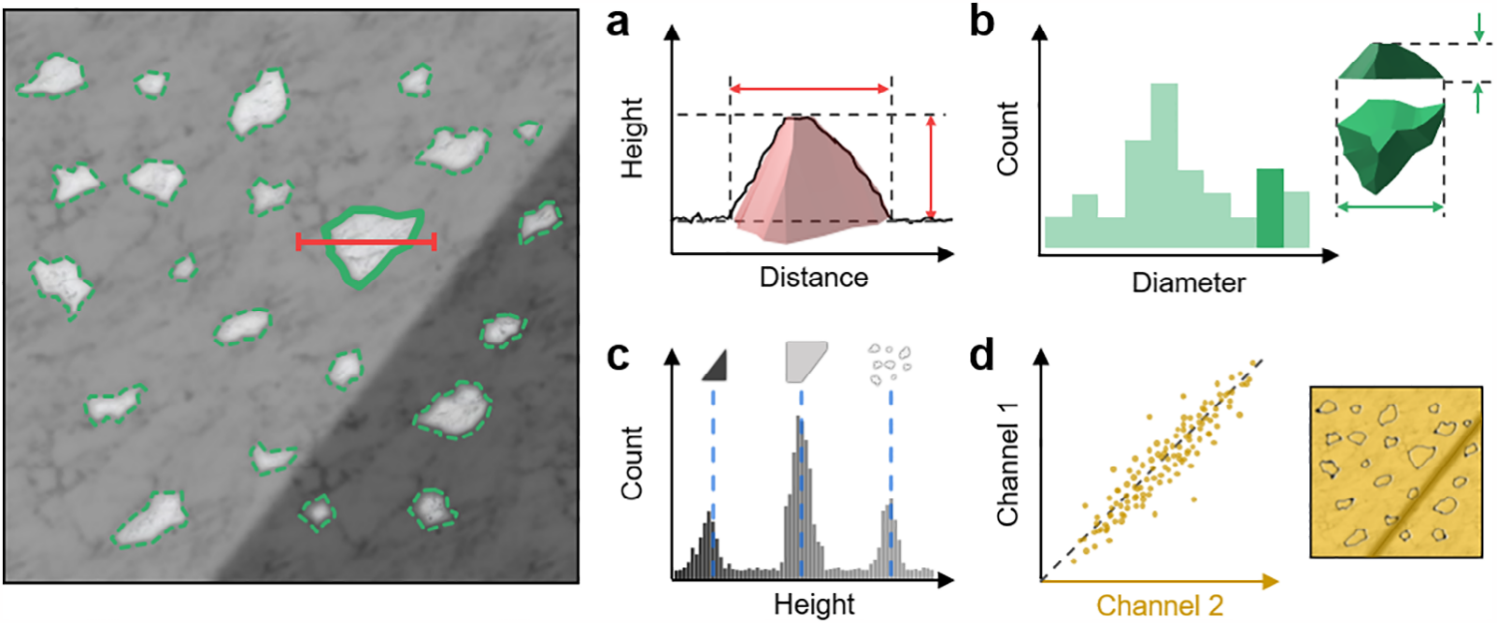
Examples of Statistical Methods. Illustration of common analysis workflows used to interpret AFM height data, which can also be applied to other types of AFM data. a) Line Profiling: A height profile is extracted along the red line shown in the left image, providing height variations across the selected region. b) Particle Analysis: A distribution plot of particle diameters highlights the bar corresponding to the particle outlined in bold green. Individual features, such as perimeter, maximum height, width, area, and volume, can also be quantified. c) Histogram Analysis: The height distribution histogram reveals distinct peaks, representing different components within the scanned area. d) Pearson Correlation: A correlation plot between two channels of a single AFM image demonstrates the relationship between measured parameters.

Line profiling involves drawing a line across a defined region of an AFM image and quantitatively analyzing height variations (or other target properties, though height is the focus here as a broadly applicable concept) (**Figure** **6**a). This technique provides clear cross‐sectional information about features of interest, such as surface roughness, distinctive topographical elements, step structures, or patterned regions. It is particularly effective for detecting subtle height variations that may be overlooked in two‐dimensional representations.

Most often, height values along a selected line are displayed as a graph upon performing line profiling, allowing the detection of minor steps, protrusions, or grooves. Determining quantitative parameters such as average height or feature width within a given region is also possible. Consequently, line profiling can be utilized in various contexts, including examining nanomaterials with hierarchical structures,^[^ ^97^, ^98^, ^99^, ^100^ ^]^ patterning semiconductor devices,^[^ ^101^, ^102^ ^]^ analyzing fibrillar structures in biological materials,^[^ ^103^, ^104^ ^]^ and characterizing active sites on catalyst surfaces.^[^ ^105^, ^106^ ^]^

#### 4.2.2 Particle Analysis

Unlike SEM, which primarily provides surface morphology with limited height information, AFM visualizes material shapes by measuring their height, volume, and roughness with picometer‐level precision. This capability is particularly advantageous for analyzing irregular or nanoscale particles, where precise dimensional data is required for understanding their behavior in applications such as catalyst,^[^ ^107^, ^108^, ^109^ ^]^ drug delivery,^[^ ^110^, ^111^ ^]^ or battery materials.^[^ ^112^ ^]^

Moreover, AFM allows for the extraction of statistical data from particle‐based analysis. By treating each particle as a distinct dataset, users can obtain distribution statistics such as particle size, height variation, or density across the scanned surface (Figure 6b). This quantitative analysis is often more detailed compared to other imaging techniques, making AFM especially valuable in studies requiring the understanding of particle characteristics or their effects on the material's overall properties.^[^ ^113^, ^114^ ^]^ Additionally, the versatility of AFM in operating under ambient or liquid conditions further enhances its ability to analyze soft or delicate particles without significant sample preparation.^[^ ^115^, ^116^, ^117^ ^]^

#### 4.2.3 Histogram Analysis

A histogram provides a comprehensive overview of the surface's height distribution (or any other target property) by plotting the frequency of observed values for each AFM image. Histograms are particularly effective for analyzing materials with bimodal or multi‐modal characteristics, where distinct peaks correspond to different components, allowing distribution‐based numerical analysis.

The strength of histogram analysis lies in its simplicity and versatility. It can reveal subtle variations in surface properties and, for height data, is especially useful for identifying features such as step edges, terraces, or nanoscale coatings (Figure 6c). Additionally, histogram analysis is inherently robust against visual artifacts, relying solely on numerical data to ensure accuracy in quantifying key surface metrics. For applications such as thin‐film characterization, histogram analysis of height data enables rapid assessments of uniformity, roughness, and thickness variations, providing valuable data for optimizing material performance in semiconductors,^[^ ^118^, ^119^, ^120^ ^]^ coatings,^[^ ^121^, ^122^ ^]^ and nanostructures.^[^ ^123^ ^]^

#### 4.2.4 Pearson Correlation

AFM generates a variety of data channels, including height, phase, amplitude, and electrical properties, depending on the selected imaging mode. In this context, Pearson correlation allows users to statistically assess the relationships between these datasets (Figure 6d). For instance, correlating height data with phase data can help determine whether specific topographical features correspond to variations in material properties, such as stiffness or adhesion.

In these applications, the key strength of Pearson correlation is its ability to provide a quantitative measure of relationships that might not be visually apparent. The capability is particularly beneficial for studying complex systems, such as composite materials or heterogenous surfaces, where multiple properties interact at the nanoscale.^[^ ^124^ ^]^ Furthermore, the method can validate hypotheses by identifying statistically significant connections between imaging parameters. For example, in battery research, a strong correlation between surface roughness and electrical conductivity might suggest pathways for optimizing electrode performance.^[^ ^125^ ^]^ This quantitative linkage between surface morphology and material functionality, achieved using Pearson correlation, enhances the interpretative power of AFM imaging.

### 4.3 Multiscale‐Multimodal Methodologies

**Figure 7:**
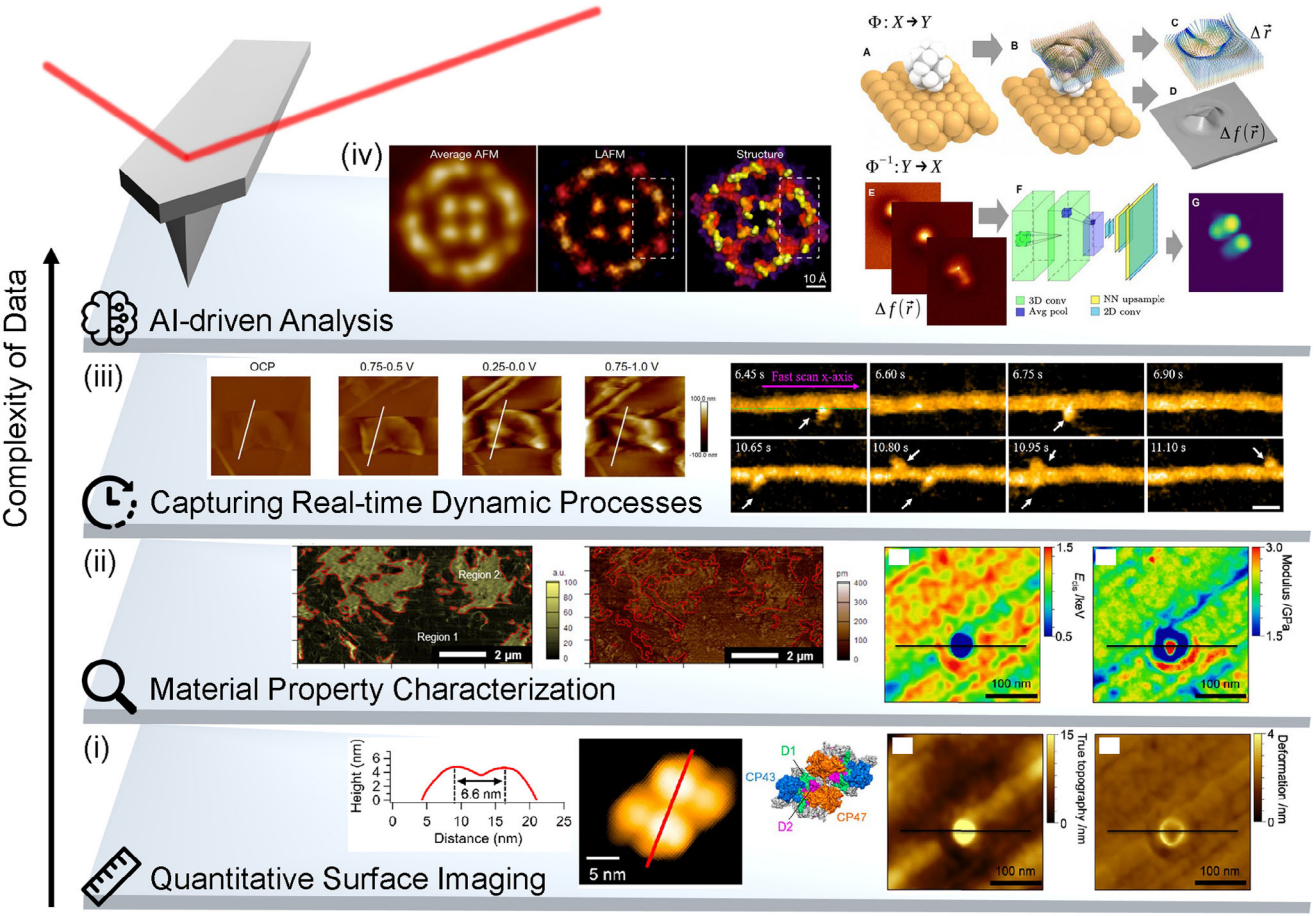
Varied Levels of Complexity in AFM Characterization. The figure demonstrates the escalating complexity of data and insights obtained through AFM, organized into four analytical layers: i) Quantitative surface imaging: The height image of multiprotein complexes and topography/deformation mapping of a nanoparticle/epoxy interface demonstrate how AFM captures precise nanoscale surface details. Reproduced with permission.^[^ ^126^ ^]^ Copyright 2020 American Chemical Society. Reproduced with permission.^[^ ^127^ ^]^ Copyright 2022 American Chemical Society. ii) Material property characterization: Electrochemical strain and friction images of a battery composite anode, along with dissipated energy (*E*_dis_) and elastic modulus images of the nanoparticle/epoxy interface from (i), exemplify AFM's role in evaluating material properties. Reproduced with permission.^[^ ^128^ ^]^ Copyright 2020 American Chemical Society. iii) Capturing real‐time dynamic processes: Operando electrochemical‐AFM images of graphite anodes and sequential images showing myosin binding to actin illustrate AFM's capability for monitoring real‐time dynamics of materials and systems. Reproduced with permission.^[^ ^129^ ^]^ Copyright 2020 American Chemical Society. Licensed under CC BY 4.0. Reproduced with permission.^[^ ^130^ ^]^ Copyright 2021 American Chemical Society. iv) AI‐driven analysis: Localization AFM applied to protein structures, revealing single amino acid resolution, is shown alongside a schematic depicting deep learning techniques for predicting molecular structures using molecule‐functionalized tips. Reproduced with permission.^[^ ^131^ ^]^ Copyright 2021 Springer Nature. Also shown is a schematic illustration of how deep learning can be applied to predict molecular structures, using molecule‐functionalized tips. Reproduced with permission.^[^ ^132^ ^]^ Copyright 2021 AAAS.

AFM is often regarded as a technique primarily used for quantitative visualization of surface topography. This perception, however, can underestimate the broader analytical capabilities enabled by advanced image processing and analysis techniques (**Figure** **7**). The evolution of AFM from simple topographical imaging to sophisticated multiscale‐multimodal analysis represents one of the most significant advancements in materials characterization over the past decade.

Figure 7 illustrates this transformation through four progressively sophisticated analytical layers, each representing a distinct information extraction and integration level. This framework helps researchers conceptualize how AFM data can be leveraged beyond basic imaging to generate deeper insights into materials.

The first level in this analytical hierarchy, Quantitative Surface Imaging (Figure 7i), forms the foundation of AFM analysis but already offers more than simple visualization. Modern AFM systems provide calibrated height measurements with subnanometer precision, enabling rigorous morphological characterization. Researchers can use statistical validity to extract standardized metrics for feature dimensions, surface roughness parameters, and spatial distributions. Unlike SEM or optical microscopy, AFM inherently provides three‐dimensional quantitative data. This enables morphological measurements that can be directly correlated with material performance metrics across multiple studies and research groups. The multiprotein complex imaging shown in the left of Figure 7i demonstrates how modern AFM can resolve individual protein subunits with precision that allows for quantitative analysis of their dimensions and spatial arrangement.^[^ ^126^ ^]^ Similarly, the nanoparticle/epoxy interface mapping reveals detailed surface features that form the foundation for more sophisticated analyses (right of Figure 7i).^[^ ^127^ ^]^

Building upon this foundation, the second analytical level, Material Property Characterization (Figure 7ii), leverages AFM's unique ability to map multiple physical properties alongside topography simultaneously. This multimodal approach reveals structure‐property relationships that would remain hidden in conventional imaging. The electrochemical strain and friction images of battery anodes shown in Figure 7ii (left) reveal how local mechanical and electrochemical properties vary across the electrode surface, providing crucial information about battery performance mechanisms.^[^ ^128^ ^]^ Similarly, the dissipated energy and elastic modulus maps of the nanoparticle/epoxy surface (right of Figure 7ii) demonstrate how AFM can differentiate between materials with similar topography but different mechanical properties, enabling nanoscale identification of phases and interfaces.^[^ ^127^ ^]^

The third level, Capturing Real‐time Dynamic Processes (Figure 7iii), addresses one of AFM's traditional limitations—temporal resolution. AFM's slow scanning speeds historically constrained its application to static or slowly evolving systems. Recent technological advances in high‐speed AFM have significantly mitigated this limitation. Furthermore, unlike electron‐based microscopy techniques that typically require vacuum environments, AFM's flexible analytical environment permits in‐situ imaging during chemical reactions, electrochemical processes, and biological interactions under native conditions.

The operando electrochemical‐AFM images of graphite anodes in Figure 7iii (left) reveal the progressive formation and evolution of the solid electrolyte interphase layer during battery cycling, capturing transient states that would be inaccessible through conventional post‐mortem analysis. Miller et al. demonstrated this approach by visualizing the solid electrolyte interphase (SEI) formation on graphite battery electrodes during electrochemical cycling.^[^ ^129^ ^]^ Their work captured SEI growth visually and quantified dynamic changes in thickness and line profiles, providing unprecedented insights into battery degradation mechanisms. The sequential myosin‐actin binding images in biological systems in Figure 7iii (right) demonstrate how high‐speed AFM can capture biomolecular interactions with millisecond temporal resolution, enabling direct visualization of protein function rather than inferring it from static structures.^[^ ^130^ ^]^

Integrating in‐situ capabilities with quantitative analysis represents a transformative advance in materials characterization. Rather than inferring dynamic processes from before‐and‐after snapshots, researchers can now directly observe and measure transformation pathways, reaction interfaces, and transient states—information critical for understanding mechanisms that control material performance and degradation. These dynamic observations bridge the structural and functional understanding gap, allowing researchers to connect nanoscale mechanisms with system‐level behaviors.

The fourth and most advanced analytical level, AI‐driven Analysis (Figure 7iv), represents the cutting edge of AFM methodology. Despite AFM's strengths, the data can be affected by artifacts and noise introduced during lengthy scans or by tip‐sample interactions. While earlier image processing methods had limited success in resolving these challenges, recent advances in machine learning and artificial intelligence offer robust new solutions.

The localization AFM image of protein structures in Figure 7iv (left) shows how AI algorithms can extract information from thousands of slightly different AFM images to achieve single‐amino‐acid resolution—a level of detail previously thought impossible with AFM. Scheuring et al. pioneered this approach with AI‐driven localization AFM to build high‐resolution structural maps of biomolecules.^[^ ^131^ ^]^ By combining thousands of subtly different AFM images of similar structures with advanced statistical learning algorithms, they achieved resolution down to the single amino acid level, a capability previously thought impossible with AFM. This approach enhances resolution and enables detailed molecular dynamics analysis from what would traditionally be considered noisy or artifact‐prone data.

Similarly, the molecular structure prediction illustration in Figure 7iv (right) demonstrates how deep learning transforms AFM from a measurement tool to a predictive platform, where neural networks trained on correlations between AFM images and known structures can interpret complex molecular configurations from relatively simple AFM data. Foster et al. applied deep learning to AFM datasets for automated 3D molecular structure predictions.^[^ ^132^ ^]^ Their approach demonstrates how AI can streamline the analytical process while extracting information beyond what human analysts might identify. By training neural networks on correlations between AFM images and known molecular structures, they developed systems capable of “reading” complex structural information from relatively simple AFM data inputs—effectively teaching computers to interpret AFM data in ways that expand human analytical capabilities. These AI‐enhanced approaches fundamentally change the relationship between measurement and interpretation, allowing researchers to extract meaningful data from previously unusable, noisy, or ambiguous AFM images.

These four analytical levels collectively illustrate the transformation of AFM from a basic imaging tool into a versatile analytical platform. The progression through these four analytical levels—from quantitative imaging to property mapping to dynamic observation to AI‐enhanced interpretation—represents a paradigm shift in how AFM contributes to materials research. Each level builds upon the previous ones, creating an integrated framework that maximizes the information extracted from nanoscale measurements. AFM images inherently contain rich information about topography and mechanical, chemical, and structural features across multiple length scales. To fully exploit this content, researchers must adopt integrated approaches that combine advanced image processing, careful instrument calibration, multimodal measurement strategies, in‐situ imaging capabilities, and AI‐based analytical frameworks.

AFM transcends its conventional imaging role by implementing this multilevel analytical approach, providing quantitative insights that guide the design, synthesis, and evaluation of advanced materials across multiple length scales and time domains. This transformation represents not simply an incremental improvement in microscopy but a fundamental shift in how we characterize and understand material systems, enabling researchers to extract previously inaccessible information from AFM measurements and bridge the gap between nanoscale structures and macroscale properties.

## 5 AFM for Collaborative Research

### 5.1 Versatility of AFM

Unlike other surface analysis techniques, such as SEM, X‐ray photoelectron spectroscopy (XPS), and secondary ion mass spectrometry (SIMS), which typically require high‐vacuum conditions, AFM operates without these constraints (see **Table** **1**). High‐resolution imaging and multi‐modal measurements are feasible in various environments, whether ambient, vacuum, or liquid. This versatility allows AFM to provide detailed surface morphology and physical property data (mechanical, electrical, or magnetic) at the nanoscale while minimizing the risk of sample damage from high‐energy electron beams.

**Table 1:** AFM versus other surface analysis techniques.

Technique	Resolution	Sample damage	Measurable properties	Surface analysis depth	Vacuum Requirement	In‐situ analysis	Refs.
AFM	Sub‐nanometer scale	Subtle	Surface topography Numerous properties, depend on modes (e.g., Mechanical, electrical, magnetic, piezoresponse, wear, friction, force spectroscopy)	Sub‐nanometer scale	None required	Available	[214, 215, 216, 217, 218, 219, 220, 221, 222, 223, 224]
SEM	Nanometer scale	Moderate	Surface morphology Crystal structure Chemical composition (with EDS)	Nanometer scale	Low‐vacuum (10^−3^–10^−5 ^Torr)	Challenging	[225, 226, 227, 228, 229, 230, 231, 232, 233]
TEM	Sub‐nanometer scale	Profound	Nanostructure imaging Atomic structure Crystal structure Chemical composition (with EDS)	Sub‐nanometer scale	Hig vacuum (10^−5^–10^−7^ Torr)	Challenging	[234, 235, 236, 237, 238, 239, 240, 241]
STEM	Sub‐nanometer scale	Moderate	Nanostructure imaging Atomic structure Crystal structure Atomic composition (with EDS)	Sub‐nanometer scale	High vacuum (10^−5^–10^−7^ Torr)	Challenging	[242, 243, 244, 245, 246, 247, 248, 249, 250]
XPS	Micrometer scale	Moderate	Chemical composition Chemical state Depth profiling Elemental quantification	Sub‐micrometer scale	Ultra‐high vacuum (10^−9^–10^−11^ Torr)	Challenging	[251, 252, 253, 254, 255, 256]
SIMS	Sub‐micrometer scale	Profound	Chemical composition Isotopic analysis Depth profiling Secondary ion identification	Nanometer scale	Ultra‐high vacuum (10^−9^–10^−11^ Torr)	Challenging	[257, 258, 259, 260, 261, 262]
Raman Spectroscopy	Nanometer scale	Subtle	Chemical composition Molecular vibration Crystallinity Stress and Strain Phase transition	Micrometer scale	None required	Available	[263, 264, 265, 266, 267, 268, 269]
FT‐IR	Micrometer scale	Subtle	Chemical composition Molecular structure Phase transition	Micrometer scale	None required	Available	[270, 271, 272, 273, 274, 275, 276]
Ellipsometry	Sub‐nanometer scale	Negligible	Surface topography Film thickness Optical properties	Sub‐nanometer scale	None required	Available	[277, 278, 279, 280, 281, 282, 283, 284]

In terms of resolution, AFM typically achieves lateral resolution in the range of 1–10 nm and vertical resolution below 1 nm, which is comparable to or better than SEM for certain applications, and is particularly advantageous for non‐conductive or soft materials.^[^ ^133^, ^134^, ^135^ ^]^ Although scanning tunneling microscopy (STM) can provide atomic resolution, it is restricted to conductive surfaces under highly controlled environments.^[^ ^2^ ^]^ Super‐resolution optical microscopy techniques, such as STED and PALM, offer ≈20–50 nm resolution but are limited to fluorescently labeled samples, primarily in biological systems.^[^ ^136^, ^137^ ^]^ These comparative advantages highlight AFM's broad applicability across materials and operating conditions. It is important to note that such performance metrics can vary depending on instrument configuration, mode of operation, and sample preparation.

Furthermore, the analytical capabilities of AFM extend beyond basic imaging, enabling the quantification of material properties in multiple dimensions, including wear, friction, and time or frequency‐dependent properties. This feature is particularly useful for examining complex materials such as biomaterials, polymers, and nanostructures. Combining high adaptability, non‐destructive imaging, and precise measurement has established AFM as an essential tool in materials research that supports comprehensive analyses across various scientific fields (see Section 6).

### 5.2 Combining AFM with Complementary Techniques

While AFM offers distinct advantages, users from diverse disciplines and backgrounds may face challenges when using it in isolation. To overcome these limitations and improve research outcomes, it is essential to integrate AFM with complementary analytical techniques. This integrated approach provides more comprehensive data, enhancing the understanding of complex systems. This section emphasizes the value of combining AFM with other methods, illustrating how such integrative strategies can lead to more thorough and multidimensional analyses.

The case studies selected for this section are drawn from recent high‐impact publications in leading journals based on citation metrics. We prioritized studies published in well‐regarded journals to ensure the inclusion of rigorous research that demonstrates the effective use of AFM in conjunction with other analytical techniques. These studies were categorized according to the specific objectives for which AFM was applied.

**Table** **2** summarizes the key findings of these studies, illustrating AFM's significant role in modern scientific research.^[^ ^138^, ^139^, ^140^, ^141^, ^142^, ^143^, ^144^, ^145^, ^146^, ^147^, ^148^, ^149^, ^150^, ^151^, ^152^, ^153^, ^154^, ^155^, ^156^, ^157^, ^158^, ^159^, ^160^, ^161^ ^]^ By integrating AFM with complementary analytical methods, researchers can extend the boundaries of material, biological, and catalytic sciences. These synergies enhance resolution and precision and provide multidimensional insights that are challenging to achieve with a single technique.

**Table 2:** Cases of integrated approaches. Acronyms of techniques are listed in the glossary with their full names.

AFM mode	Integrated techniques	Analysis objectives	Refs.
Nanoindentation	SEM	To characterize the coating properties and mechanical behavior of polyelectrolyte complex layers on Zn.	[138]
Topography	SEM, HPGPC, FT‐IR, HPLC‐ELSD, GC‐MS, NMR	To elucidate the molecular composition and structure of a novel water‐soluble polysaccharide.	[139]
Topography	FESEM, XRD, Raman spectroscopy, XPS	To analyze dopant‐induced variations in structural and surface properties of thin films.	[140]
Topography	TEM, Zeta potential measurements, TGA, XPS, UV‐vis Fluorescence spectroscopy, Raman spectroscopy, FT‐IR	To characterize graphene oxide nanosheets, ensuring they are of tightly defined size and free from metallic or other elemental contamination, residues, or indicators of bacterial contamination.	[141]
Topography	2D‐IR nanospectroscopy	To investigate molecular structures, anharmonicity, and energy transfers in heterogeneous nano‐materials.	[142]
Topography	SEM, ATR‐FTIR, EDX, Contact angle measurement	To examine the morphological, structural, and compositional differences between molecularly imprinted polymers and non‐imprinted sensors.	[143]
Topography	QCM‐D, Ellipsometry	To analyze the layer‐by‐layer growth behavior, stability, and surface morphology of chitosan/alginate films.	[144]
Topography	SEM, CLSM	To investigate bacteria photo‐inactivation process.	[145]
HS‐AFM	Biochemical Assays, Cryo‐ET	To investigate real‐time interactions between bacteria and antimicrobial peptides, visualizing membrane disruption mechanisms	[146]
Topography	Time‐lapse fluorescent calcium imaging, Optical microscopy, Rheology, Tribology, SRM, Patch clamping	To explore mechanosensitive proteins like Piezo, tracking mechanical responses and cell morphology in biological systems, and understand of the principles underlying oral texture perception, incorporating insights from cell/molecular biology, genetics, and sensory trials.	[147]
Topography	Rheometry	To investigate viscoelasticity changes in mice fed different diets.	[148]
AFM‐IR	SFG	To identify cobalt phthalocyanin monomers, aggregates, and c intermediates with/without cation stabilization.	[149]
Topography	UPS	To investigate valence band positions and Fermi levels of catalyst coated Si electrodes.	[150]
PiFM, KPFM	4D‐STEM	To investigate the unexplored role of N‐methylformamidinium in formamidinium lead triiodide perovskite with methylammonium chloride incorporation that induces formamidinium cation heterogeneity in formamidinium lead triiodide.	[151]
KPFM	SPV, Charge density difference analysis, First principles calculations	To confirm that the ligand molecular bridge enhances electron transfer from metal‐organic framework to Ti_3_C_2_O_x_, capturing electrons and improving charge carrier separation, which boosts photocatalytic performance.	[152]
In‐situ KPFM	Energy band calculation	To reveal that the p‐n heterojunction formed by Co_3_O_4_@FeO_x_ generates a built‐in electric field, enhancing uranium electro‐reduction.	[153]
KPFM	DFT	To verify the internal electric field and S‐scheme heterojunction formation in graphitic carbon nitride and BaFe_12_O_19_.	[154]
KPFM	DFT, In‐situ XPS, PL, EPR	To explore electronic interactions and charge migration in the ZnO/In_2_S_3_ composite.	[155]
Topography	STEM	To analyze the crystalline quality of materials.	[156]
Topography	SEM, FM, Contact angle measurements, CV, SWV, EIS	To collectively provide insights into the “dopamine‐gold nanoparticle”, “dopamine‐graphene quantum dots”, and “dopamine‐gold nanoparticle‐graphene quantum dots” structures, wettability, and electrochemical behavior for biomolecular detection.	[157]
Topography	Raman spectroscopy	To measure the thickness of CrSBr flakes.	[158]
Topography	POM, ESEM, TEM, WAXD, FT‐IR	To confirm the integration of cellulose nanocrystals within electrospun fibers.	[159]
Topography	8% PAGE, HPCE, DLS, TEM	To verify the synthesis and structure of tetrahedral framework nucleic acids‐based targeting dual‐microRNA nanobiocarriers as a delivery system.	[160]
Topography	8% PAGE, DLS, TEM	To verify the molecular weights, morphology, and hydrodynamic properties of tetrahedral framework nucleic acids and tFNA‐loaded antimicrobial peptide (t‐GL13K).	[161]

It is noteworthy that most collaborative AFM research only includes basic topography analysis using AFM. As mentioned earlier, AFM can analyze a wide range of material properties, and therefore, each analysis mode has the potential to be an excellent collaborative technique. If sufficient understanding of each AFM mode is established beforehand, the approach to analysis itself becomes a new frontier. The following chapters will discuss several points for effective AFM collaboration.

#### 5.2.1 Material and Structural Properties

In materials science, combining AFM with techniques such as electron microscopy and spectroscopy allows for a more comprehensive characterization of mechanical and structural properties. Yang et al. used AFM, SEM, and nanomechanical mapping to analyze coatings on metal surfaces, uncovering a series of representative parameters reflecting the mechanical properties, including Young's modulus, adhesion work, and dissipation ability.^[^ ^138^ ^]^ Similarly, Gong et al. employed AFM alongside high‐performance gel permeation chromatography (HPGPC), Fourier transform infrared spectroscopy (FT‐IR), and nuclear magnetic resonance (NMR) to investigate the molecular composition and structure of polymeric materials.^[^ ^139^ ^]^ Doped thin films are another area where AFM integration has shown notable potential. Ajjaq et al. combined AFM with field emission scanning electron microscope (FE‐SEM), X‐ray diffraction (XRD), Raman spectroscopy, and XPS to infer the gas‐sensing performance before and after doping. Their analysis was based on key observations such as the reduction in crystallite size from XRD, the higher aspect ratio revealed by SEM, the increased surface roughness and porosity identified via AFM, and the enhanced oxygen vacancy sites detected through XPS.^[^ ^140^ ^]^

For nanomaterials, Andrews et al. used AFM, TEM, and ultraviolet‐visible spectroscopy (UV‐Vis) to characterize the size distribution and homogeneity in graphene oxide nanosheets.^[^ ^141^ ^]^ These examples illustrate how AFM, paired with complementary methods, advances the understanding of material properties and nanoscale structures. Additionally, Xie et al. employed AFM in combination with two‐dimensional infrared (2D‐IR) nanospectroscopy to study molecular structures and energy transfers in nanostructures, demonstrating the versatility of AFM for structural and compositional studies.^[^ ^142^ ^]^ Cetinkaya et al. employed AFM, SEM, contact angle measurements, Fourier transform infrared spectrophotometry attenuated total reflection (FTIR‐ATR), and energy dispersive X‐ray spectroscopy (EDX) to investigate the surface morphology, thickness, and chemical composition of imprinted and non‐imprinted sensors based on an antiviral compound.^[^ ^143^ ^]^ Their findings showed that the imprinted sensor had a rougher, more irregular surface with higher hydrophilicity than the non‐imprinted version. Moreover, Tidim et al. demonstrated the usefulness of combining AFM with ellipsometry and quartz crystal microbalance with dissipation (QCM‐D) to analyze the layer‐by‐layer growth, stability, and surface morphology of chitosan/alginate films, as well as phage deposition into multilayers.^[^ ^144^ ^]^

#### 5.2.2 Biological Mechanisms and Interactions

In biological research, AFM is important in probing molecular interactions and cellular processes. AFM also supports studying biological systems, particularly in understanding cellular responses and membrane interactions. Nayak et al. integrated AFM with confocal microscopy and SEM to observe oxidative damage in bacterial cells during photocatalytic treatments, linking reactive oxygen species to membrane disruption.^[^ ^145^ ^]^ Furthermore, Ismail et al. employed high‐speed AFM (HS‐AFM) in conjunction with cryo‐electron tomography (Cryo‐ET) to investigate real‐time interactions between bacteria and antimicrobial peptides, providing a detailed analysis of membrane disruption pathways.^[^ ^146^ ^]^ Meanwhile, Koehler et al. integrated AFM with time‐lapse fluorescent calcium imaging and super‐resolution microscopy to investigate mechanosensitive proteins such as the Piezo family, advancing the understanding of biological systems and cellular responses.^[^ ^147^ ^]^ Fan et al. used AFM in combination with rheometry to investigate the viscoelastic properties of biological materials under varying conditions.^[^ ^148^ ^]^ They observed that changes in the extracellular matrix were associated with increased viscoelasticity in cases of metabolic disorders and specific dietary conditions. Such integrative approaches shed light on dynamic biological processes.

#### 5.2.3 Catalytic and Photocatalytic Systems

For catalytic systems, AFM integration with spectroscopic and imaging methods provides critical information on electronic properties and charge transfer dynamics. Zhu et al. combined AFM‐IR with surface‐selective sum frequency generation (SFG) spectroscopy to identify catalytic intermediates and reaction pathways, such as cobalt‐based monomers and carbon monoxide stabilization in catalytic systems.^[^ ^149^ ^]^ Balog et al. employed AFM and ultraviolet photoelectron spectroscopy (UPS) to analyze valence band structures and Fermi levels in catalyst‐coated electrodes.^[^ ^150^ ^]^ In photocatalysis, Lim et al. combined photo‐induced force microscopy (PiFM), KPFM, and four‐dimensional scanning transmission electron microscopy (4D‐STEM) to study cation heterogeneity and charge transfer dynamics in perovskite systems.^[^ ^151^ ^]^ Similarly, Shen et al. employed KPFM, surface photovoltage (SPV) signal measurements, and first‐principles calculations to better understand charge transfer dynamics in photocatalytic systems.^[^ ^152^ ^]^ These studies demonstrate the utility of AFM in exploring catalytic mechanisms.

#### 5.2.4 Integration with Computational Models

Integrating AFM with computational models has proven valuable for investigating material behavior. For example, Zhou et al. combined energy band calculations with in‐situ KPFM to show that the p‐n heterojunction formed by Co_3_O_4_@FeO_x_ generates a built‐in electric field, enhancing uranium electro‐reduction.^[^ ^153^ ^]^ This illustrates the powerful synergy between AFM and computational models for understanding complex electrochemical processes. Similarly, Zhou et al. utilized KPFM along with density functional theory (DFT) to confirm the formation of internal electric fields and charge transfer paths in the g‐C_3_N_4_/BaFe_12_O_19_ heterojunction, further highlighting the potential of integrating AFM with computational models.^[^ ^154^ ^]^ Ai et al. investigated the performance enhancement of the ZnO/In_2_S_3_ S‐scheme heterojunction using KPFM, XPS, and DFT calculations, providing a detailed analysis of charge migration, electronic interactions, and charge separation efficiency. The heterojunction generates an internal electric field that promotes electron‐hole separation, highlighting the importance of efficient charge separation and interfacial interactions.^[^ ^155^ ^]^

Beyond DFT‐based approaches, recent studies have demonstrated the integration of AFM with alternative simulation and numerical methods such as machine learning (ML) and quantitative structure–activity relationship (QSAR) models to predict nanomaterial behavior in biological systems.^[^ ^162^ ^]^ For instance, in the context of nanobiology, nanoscale descriptors obtained through AFM can be coupled with ML‐driven nano‐QSAR workflows to predict properties like biocompatibility, cellular uptake, and toxicity. These integrated approaches enable data‐driven insights by correlating nanoscale surface features with biological interactions, enhancing the predictive power and interpretability of AFM measurements in complex environments. Such developments underscore the versatility of AFM when combined with a broader spectrum of computational techniques, extending its utility beyond conventional electronic structure analyses.

### 5.3 Custom AFM Probe Designs for Specialized Applications

Recent developments in AFM probe design have focused on overcoming the intrinsic limitations of conventional silicon‐based probes by employing novel materials, geometries, and fabrication techniques tailored for specific imaging and sensing requirements. A prominent example is the utilization of 3D nanoprinting via direct laser writing to fabricate monolithic polymer‐based cantilevers with tunable mechanical properties and complex geometries. These 3D‐printed probes offer an order of magnitude higher bandwidth in intermittent contact mode and enable modal engineering for nonlinear tip–sample interactions, thereby expanding the functional landscape of AFM beyond the capabilities of traditional micromachined silicon probes.^[^ ^163^ ^]^

Graphene‐coated polymer probes represent another innovative direction, wherein graphene is conformally grown on prepatterned substrates and subsequently transferred to SU‐8 probes. These hybrid structures exhibit enhanced wear resistance and electrical conductivity, making them suitable for C‐AFM applications and studies of graphene–graphene interactions at the nanoscale.^[^ ^164^ ^]^ In parallel, fully metallic AFM probes have been fabricated via electrodeposition into molds formed by indentation and photolithography. These all‐metal nickel probes, which span a wide stiffness range (20–2800 N m^−1^), show promise in tribological studies due to their excellent mechanical robustness and frictional performance.^[^ ^165^ ^]^

Custom probes integrating optical elements have also been introduced to improve the throughput and resolution of AFM imaging. For instance, microlens‐coupled AFM probes, equipped with focused ion beam (FIB)‐deposited tips, have demonstrated high‐throughput optical‐AFM correlative imaging. This setup achieves multiscale spatial resolution (from micrometers to nanometers) while preserving force feedback control, significantly enhancing the utility of AFM in semiconductor inspection.^[^ ^166^ ^]^

For three‐dimensional characterization, orthogonal cantilever probes (OCPs) have been developed to enable multimode 3D Kelvin probe force microscopy (3D‐KPFM). These custom‐designed probes incorporate nanoscale tips protruding from both the side and underside of the cantilever, enabling simultaneous 2D surface and vertical sidewall imaging. Such designs facilitate comprehensive mapping of surface potential and topography across complex 3D micro/nanostructures.^[^ ^167^ ^]^

Collectively, these custom AFM probe technologies not only address the performance bottlenecks of conventional probes but also expand the methodological and application scope of AFM, especially in fields requiring high‐resolution, high‐throughput, and multidimensional characterization.

### 5.4 Key Considerations for Collaboration

To ensure effective collaboration in AFM analysis, collaborators must follow a structured protocol addressing key preparatory steps. The process begins with clearly defining the analytical objectives, such as measuring surface roughness, height profiles, nanostructures, mechanical properties, or interfacial interactions. Collaborators are encouraged to specify the expected data outputs, including 2D/3D visualizations or quantitative metrics, ensuring alignment with the analytical goals.

Preparation of the sample is critical, encompassing thorough characterization of its material properties (e.g., composition, surface morphology, and dimensions) and careful cleaning to remove contaminants such as organic residues, particulates, or oils. Depending on the sample's sensitivity or analytical requirements, advanced cleaning methods such as ethanol rinsing, ultrasonic cleaning, plasma cleaning, or surface smoothing may be required, particularly for modes like force‐distance spectroscopy or force volume imaging that demand clean, flat surfaces for precise adhesion and interaction force measurements. Proper mounting techniques, including conductive tapes or adhesives, must also be implemented to ensure sample stability during scanning and prevent artifacts caused by movement or uneven surfaces.

The selection of AFM measurement modes (e.g., contact, tapping, or non‐contact) should be informed by the sample's properties and the intended analytical outcomes. Additionally, effective collaboration in multi‐modal analysis requires a solid understanding of diverse AFM modes and proper sample preparation techniques. Each AFM mode has specific requirements for optimal performance, such as the choice of the tip and the need for additional equipment (**Table** **3**). For instance, FMM typically requires a high‐stiffness tip and mechanical excitation to map mechanical variations, while nanoindentation AFM uses a high‐strength diamond tip for measuring hardness and elastic modulus at the nanoscale. For studying surface charge distributions or electrical properties, EFM employs a conductive‐coated tip, while MFM relies on a magnetically coated tip for high‐resolution magnetic analysis.

**Table 3:** Various AFM modes and details.

Mode	Analyzable information	Operating mode	Recommended tip choice	Special requirements	Notes	Refs.
Force Modulation Microscopy (FMM)	Mechanical property variations	Contact	High‐stiffness tip recommended	Mechanical excitation required	Suitable for comparing mechanical properties of soft and hard materials	[285, 286, 287, 288]
Lateral Force Microscopy (LFM)	Frictional properties, mechanical heterogeneity	Contact	Longer tip recommended	None	Measures frictional forces on the surface	[289, 290]
Phase Imaging AFM	Viscoelasticity, chemical composition contrast	Tapping	Standard Si or SiN tip usable	Vibration mode required	Typically used with tapping mode for phase contrast analysis	[291, 292, 293]
Force‐Distance Spectroscopy	Adhesion, attractive forces, surface energy	Contact	High‐stiffness tip recommended	None	Measures adhesion and interaction forces via force‐distance curves	[194, 294, 295, 296]
Force Volume Imaging	Local mechanical property mapping	Contact	High‐stiffness tip recommended	Mechanical mapping required	Enables mechanical mapping of specific regions	[297, 298, 299, 300]
AFM Nanoindentation	Hardness, elastic modulus, plastic deformation	Contact	High‐strength, diamond tip recommended	High‐strength probe required	Measures mechanical properties at nanoscale	[301, 302, 303, 304]
Magnetic Force Microscopy (MFM)	Magnetic field distribution, magnetic properties	Non‐contact, Tapping	Magnetically coated tip required	Magnetic coating on tip required	High‐resolution magnetic analysis	[305, 306, 307, 308, 309]
Electrostatic Force Microscopy (EFM)	Surface charge distribution, electrical properties	Non‐contact, Tapping	Conductive coated tip required	Electrically conductive tip required	Measures electrical properties on the surface	[310, 311, 312]
Nano Thermal Analysis AFM (nanoTA‐AFM)	Local thermal properties	Contact	Heat‐conductive tip required	Heat‐conductive probe required	Measures local thermal properties at specific locations	[313, 314, 315]
Electrochemical AFM (EC‐AFM)	Surface changes due to electrochemical reactions	Contact	Conductive coated tip recommended	Electrochemical cell required	Measures surface changes in electrochemical environments	[316, 317, 318, 319]
AFM with Infrared Spectroscopy (AFM‐IR)	Local chemical composition, molecular vibrations	Contact	Standard Si or SiN tip usable	Infrared light source required	Combines IR spectroscopy for chemical analysis	[320, 321, 322]
AFM with Tip‐Enhanced Raman Spectroscopy (AFM‐TERS)	Local chemical composition, molecular structure	Contact, Tapping	Metal‐coated tip (e.g., Au, Ag) required	Raman signal enhancement required	Enhances Raman signals for high‐resolution analysis	[323, 324, 325, 326, 327, 328, 329]
Piezoelectric Response AFM (PFM)	Piezoelectric properties, electromechanical response	Contact	Conductive tip required	Electrical signal application required	Measures piezoelectric and electromechanical responses	[5, 330, 331]
Amplitude‐Frequency Modulation AFM (AM‐FM AFM)	Viscoelasticity, mechanical properties	Tapping	Standard Si or SiN tip usable	Amplitude and frequency modulation required	Combines amplitude and frequency modulation for analysis	[83, 332, 333, 334, 335, 336, 337]
Terahertz AFM (THz‐AFM)	Physical/electromagnetic properties at THz frequencies	Contact, Non‐contact	Standard Si or SiN tip usable	Terahertz light source required	Measures properties in the THz frequency range	[338, 339, 340, 341]
Scanning Microwave Impedance Microscopy (SMIM)	Local electrical impedance	Contact	Microwave‐reflective tip required	Microwave signal application required	Measures electrical impedance distribution	[342, 343, 344]
Scanning Capacitance Microscopy (SCM)	Electrical doping profile, capacitance	Contact	Conductive coated tip required	Electrical signal application required	Measures doping profiles in semiconductors	[345, 346, 347, 348]
Optical and Thermal Scanning Probe Lithography (o‐SPL & t‐SPL)	Local optical and thermal patterning	Contact	Special patterning tip required (optical/thermal)	Laser or heat source required	Uses laser or heat for surface patterning	[349, 350, 351, 352]

Providing detailed information about the sample is also essential for effective collaboration. This includes its composition, substrate, thickness, coating, and relevant environmental or physical sensitivities. The required scan area, resolution, and the need for repeated measurements should also be specified. These must be communicated in advance if environmental conditions (e.g., humidity or inert gas requirements) are critical to the sample. Collaborators should verify that the experimental parameters are well‐coordinated with the analytical team, ensuring potential limitations and risks are addressed before analysis.

Post‐analysis, collaborators should review the results to confirm they meet the defined objectives. Any discrepancies or additional requirements, such as alternative scanning parameters or further analysis, should be promptly communicated. This protocol ensures that AFM measurements are precise, reproducible, and aligned with the intended research outcomes, supporting a collaborative process between clients and analytical teams.

In summary, collaborators should engage in detailed discussions with AFM operators regarding the sample's characteristics, research objectives, and appropriate AFM modes to tailor the analysis to specific needs. Such collaborative efforts foster a robust and efficient workflow, enhancing material characterization accuracy, reproducibility, and depth.

## 6 Applications of AFM in Materials Research

Aiming to help collaborators determine whether AFM fits their research needs, we now transition to an overview of the practical implementations of AFM across a range of material systems. The major material types investigated with AFM are organized into subsections, with specific techniques and methods for obtaining meaningful data discussed for each category. **Table** **4** presents a summary of these materials and subcategories. While the scope and details may have specific limitations, the purpose of this review is to help researchers identify the most relevant measurable properties in their fields. Each subsection is accompanied by a figure that illustrates representative applications and presents data along with key findings from various AFM studies.

**Table 4:** Overview of AFM applications across diverse material types.

Material type	Subcategory	Applications of AFM	Related Figure
Metals	Pure Metals and Alloys	Grain boundary characterization and morphological analysis^[^ ^168^, ^353^, ^354^, ^355^ ^]^	Figure 8
		Phase identification and spatial distribution mapping^[^ ^169^, ^356^ ^]^	
		Electrical characterization of metallic nanostructures^[^ ^170^, ^357^, ^358^, ^359^ ^]^	
		Nanostructure fabrication and patterning^[^ ^172^, ^360^, ^361^, ^362^ ^]^	
		Corrosion behavior in electrolytes^[^ ^173^, ^363^, ^364^, ^365^, ^366^ ^]^	
		Magnetic domain imaging and magnetization reversal analysis^[^ ^174^, ^367^, ^368^ ^]^	
Semiconductors	Elemental and Compound Semiconductors	Dopant profiling and carrier distribution analysis^[^ ^22^, ^369^, ^370^, ^371^, ^372^, ^373^ ^]^	Figure 9
		Defect identification and nanoscale failure diagnostics^[^ ^175^, ^374^, ^375^ ^]^	
		Nanolithography and electrode patterning^[^ ^66^, ^176^ ^]^	
		Nanoscale electrical conductivity characterization^[^ ^177^, ^376^, ^377^, ^378^ ^]^	
Ceramics	Structural Ceramics	Young's modulus and adhesion force analysis^[^ ^379^, ^380^, ^381^, ^382^ ^]^	Figure 10
		Grain boundary and microstructural analysis^[^ ^179^, ^383^ ^]^	
	Functional Ceramics	Piezoelectric domain imaging and displacement measurements^[^ ^5^, ^26^, ^384^, ^385^ ^]^	
		Ferroelectric polarization switching studies^[^ ^85^, ^386^, ^387^ ^]^	
		Domain kinetics and screening charge dynamics^[^ ^36^, ^182^, ^388^, ^389^ ^]^	
	Bio‐ceramics	Erosion and surface deformation behavior analysis^[^ ^184^, ^390^, ^391^ ^]^	
		Dielectric properties and surface charge distribution^[^ ^392^, ^393^, ^394^ ^]^	
Polymers and Soft Materials	General Polymers	Visualization and analysis of polymer chain structures^[^ ^185^, ^395^, ^396^, ^397^ ^]^	Figure 11
		Nanomechanical property mapping and mechanical characterization^[^ ^186^, ^398^, ^399^, ^400^ ^]^	
	Conductive Polymers	Electrical characterization and conductivity mapping^[^ ^187^, ^401^, ^402^, ^403^ ^]^	
	Block Copolymers	Imaging of self‐assembled nanostructures^[^ ^187^, ^404^, ^405^, ^406^, ^407^ ^]^	
	Hydrogels	Mechanical properties and swelling behavior characterization^[^ ^188^, ^408^, ^409^, ^410^ ^]^	
Biological Materials	Macromolecules, Cells, and Tissues	Structural analysis of DNA and proteins in aqueous environments^[^ ^189^, ^411^, ^412^, ^413^ ^]^	Figure 12
		Cell mechanics and adhesion^[^ ^9^, ^190^, ^414^, ^415^, ^416^, ^417^, ^418^ ^]^	
		High‐speed imaging of dynamic biological processes^[^ ^191^, ^419^, ^420^, ^421^, ^422^, ^423^ ^]^	
		Structural characterization via correlative imaging and data reconstruction^[^ ^131^, ^192^, ^424^, ^425^, ^426^ ^]^	
Nanomaterials	Nanoparticles and Quantum Dots	Growth kinetics, morphology, and size distribution analysis^[^ ^427^, ^428^, ^429^, ^430^, ^431^ ^]^	Figure 13
		Nanomanipulation and assembly^[^ ^196^, ^432^, ^433^ ^]^	
		Electrical transport studies^[^ ^197^, ^434^, ^435^ ^]^	
	Nanowires and Nanotubes	Nanowire‐based circuit construction^[^ ^198^, ^436^ ^]^	
		Mechanical properties and flexural rigidity^[^ ^437^, ^438^, ^439^ ^]^	
	2D Nanomaterials	Electrical characterization and conductivity mapping^[^ ^199^, ^440^, ^441^, ^442^, ^443^, ^444^, ^445^ ^]^	
		Mechanical strain engineering and manipulation^[^ ^200^, ^446^, ^447^, ^448^ ^]^	
		In situ intercalation studies^[^ ^201^, ^449^, ^450^, ^451^ ^]^	
Composite and Hybrid Materials	Polymer‐based Composites	Nanomechanical property mapping and dispersion analysis^[^ ^202^, ^452^, ^453^, ^454^, ^455^, ^456^, ^457^ ^]^	Figure 14
	Metal‐Organic Frameworks (MOFs)	Crystal formation and growth processes^[^ ^203^, ^458^, ^459^ ^]^	
		Mechanical stability and elastic modulus measurement^[^ ^204^, ^460^, ^461^ ^]^	
		Electrical conductivity and resistive switching behaviors^[^ ^205^, ^462^, ^463^ ^]^	
Energy Materials	Photovoltaics	Photocurrent mapping and charge distribution^[^ ^206^, ^464^, ^465^, ^466^, ^467^, ^468^, ^469^ ^]^	Figure 15
	Battery Electrodes and Electrolytes	Real‐time surface dynamics in electrochemical cycling^[^ ^129^, ^207^, ^470^, ^471^, ^472^, ^473^ ^]^ Spatial observation of ionic distribution^[^ ^27^, ^29^, ^128^, ^208^ ^]^	

### 6.1 Metals

Metals are essential in engineering and technological applications due to their mechanical strength, electrical conductivity, and resistance to various environmental factors. The study of metals at the nanoscale is especially important for understanding and enhancing these properties, as well as for tailoring them for specialized applications. In these investigations, AFM offers high‐resolution imaging, quantification of electrical properties, and analysis of mechanical properties. Its ability to operate in diverse environmental conditions extends its applications to nanoscale fabrication and corrosion analysis. The following examples highlight such diverse applications of AFM in metals research.

**Figure 8:**
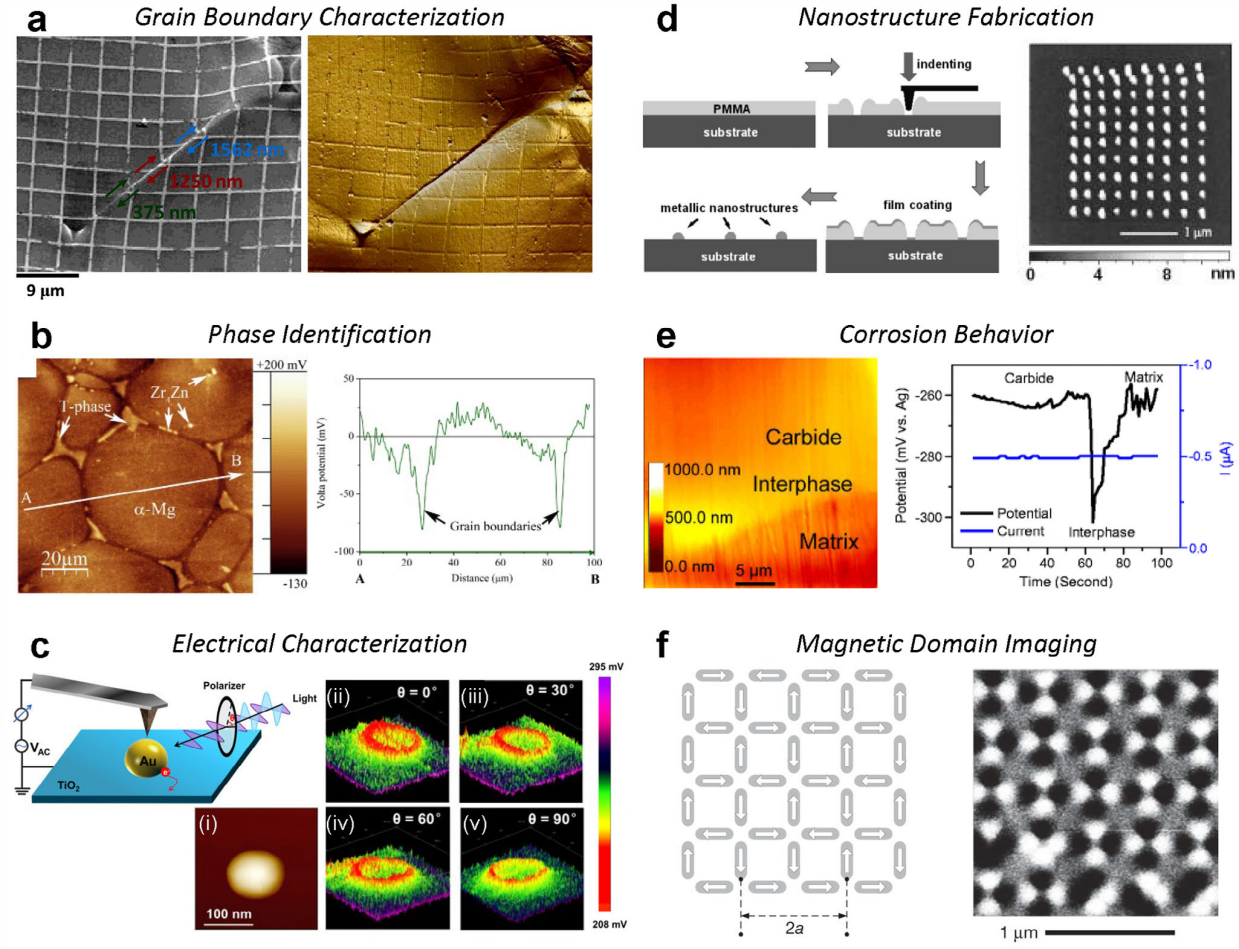
Applications of AFM in Metals. a) SEM and AFM images showing in‐plane grain boundary sliding in titanium, with fiducial grid distortions indicating local slip activity. Reproduced with permission.^[^ ^168^ ^]^ Copyright 2016, Elsevier. b) Surface potential map and line‐profile showing potential variations across the metal matrix in a rare‐earth magnesium alloy. Reproduced with permission.^[^ ^169^ ^]^ Copyright 2010, Elsevier. c) KPFM surface potential images showing the impact of light polarization on Au/TiO_2_ nanoparticles, showing height (i) and charge distribution at different polarization angles (ii‐v). Reproduced with permission.^[^ ^170^ ^]^ Copyright 2020, Wiley‐VCH GmbH. d) Experimental schematic and AFM image of a gold nanodot array fabricated using AFM‐based nanomachining and lift‐off. Reproduced with permission.^[^ ^171^ ^]^ Copyright 2004, American Vacuum Society. e) AFM image and line‐sections showing local potential and current variations indicative of corrosion near the interface between the primary carbide and matrix in high‐chromium cast iron. Reproduced with permission.^[^ ^173^ ^]^ Copyright 2015, Springer Nature. f) Schematic illustration and MFM image of an artificial square spin ice, showing magnetic poles (right) and magnetic moments (left) of ferromagnetic nanostructures. Reproduced with permission.^[^ ^174^ ^]^ Copyright 2013, Springer Nature.

AFM, in conjunction with SEM, has been utilized to investigate grain boundary characteristics in titanium (**Figure** **8**a). Fiducial grids, fabricated using electron beam lithography, enabled precise analysis of grain boundary sliding through local distortions. While the image depicts an instance of in‐plane sliding, AFM also facilitated the measurement of ledge heights formed by out‐of‐plane sliding in the mentioned study, a capability not achievable with standard SEM‐based measurements.^[^ ^168^ ^]^

KPFM also allows for phase identification in microstructural systems, and this can be further adapted for use with environmental modifications. Figure 8b shows KPFM applied for phase characterization in a rare‐earth magnesium alloy, where the surface potential map reveals potential differences between the primary metal matrix and secondary phases, providing insight into its micro‐galvanic interactions.^[^ ^169^ ^]^ An example of KPFM's environmental adaptability is demonstrated by a light‐irradiated KPFM study for the electrical characterization of metal/semiconductor systems, as shown in Figure 8c. With the investigation of the surface potential of single Au nanoparticles deposited on a TiO_2_ surface under polarized right, the relationship between polarization direction and local charge is revealed, elucidating plasmon‐induced charge transfer mechanisms.^[^ ^170^ ^]^

The versatility of AFM also extends to nanostructure fabrication and corrosion studies. Figure 8d depicts the creation of metallic nanodots through AFM‐based nanomachining. In this process, precise grooves or holes are etched into a resist‐coated silicon substrate, which are subsequently developed into metallic structures through deposition and lift‐off.^[^ ^171^, ^172^ ^]^ In corrosion research, electrochemical AFM (EC‐AFM) has been instrumental in investigating localized corrosion rates. In Figure 8e, EC‐AFM was applied to examine the interface between the primary carbide and matrix in high‐chromium cast iron, revealing higher anodic activity near the interface. These experiments are typically conducted with an electrochemical cell that connects the tip and sample to a potentiostat/galvanostat.^[^ ^173^ ^]^

Lastly, MFM has been utilized to directly observe magnetic domains in artificial spin ice systems (Ni_80_Fe_20_).^[^ ^174^ ^]^ In this study, MFM captured images of the magnetic moments of ferromagnetic nanostructures arranged in square lattices (Figure 8f), revealing crystallites of magnetic charges. This technique can be broadly applied to other metallic systems that exhibit magnetic domains as well.

### 6.2 Semiconductors

In semiconductor research, key parameters such as dopant concentration, contact resistance, and electric behavior at the interfaces are critical factors that affect the performance of semiconductor devices. In this context, AFM‐based techniques provide high‐resolution imaging and electrical characterization, allowing users to investigate and manipulate materials with detailed precision. These methods are valuable for both facilitating fundamental research and addressing challenges in device fabrication and failure analysis, where nanoscale characterization is necessary.

**Figure 9:**
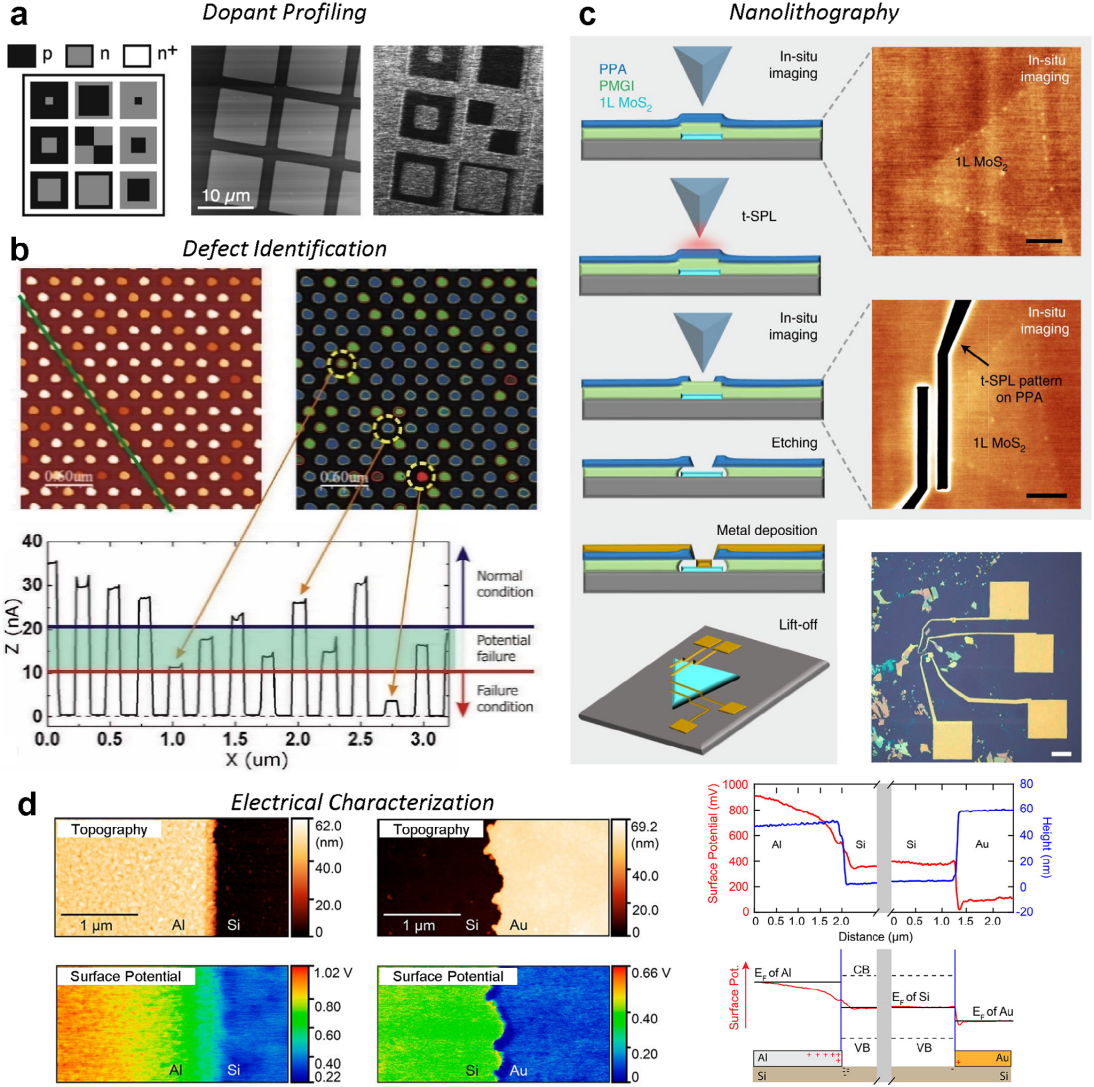
Applications of AFM in semiconductors. a) Schematic of doping patterns on a silicon test sample, an AFM topographic image and a corresponding SCFM image indicating p‐type regions as dark areas. Reproduced with permission.^[^ ^22^ ^]^ Copyright 2002, AIP Publishing. b) C‐AFM image obtained on a DRAM cell exhibiting resistive behavior, shown next to a corresponding color map where contact points are categorized as blue (normal), green (marginal), and red (failure) in terms of current levels. Line profile shows the current levels with failure criteria. Reproduced with permission from refererence.^[^ ^175^ ^]^ Copyright 2013, Springer Nature. c) t‐SPL enables in‐situ topographic imaging and high‐resolution patterning on a two‐layer polymer stack. Subsequent chemical etching, deposition, and lift‐off result in clearly defined electrodes. Reproduced with permission.^[^ ^176^ ^]^ Copyright 2019, Springer Nature. d) Surface potential mapping of metal‐semiconductor boundaries. The Al/Si boundary appears gradual and continuous, whereas the Si/Au boundary is sharp and well‐defined. The bottom right schematic illustrates the relative band positions. Reproduced with permission.^[^ ^177^ ^]^ Copyright 2020, American Chemical Society.

Traditional methods for characterizing semiconductors, such as spreading resistance profiling and SIMS, often lack the ability to probe functional properties at the sub‐device scale. In contrast, SCM offers a solution by measuring capacitance changes between the AFM tip and sample, allowing for surface mapping of dopant concentrations. **Figure** **9**a shows an application of SCFM, a variation of SCM that eliminates the need for an external capacitance sensor. In this example, differential capacitance (∂C/∂V) is mapped on a silicon sample with patterned n‐type and p‐type regions, providing a clear contrast between doped areas. This approach enables visualization of doping distributions at the nanoscale.^[^ ^22^ ^]^

C‐AFM is also useful for failure analysis in semiconductor materials, particularly for non‐destructive evaluation of contact behavior. In the study shown in Figure 9b, dynamic random‐access memory (DRAM) cells exhibiting resistive failure display varying current levels for self‐aligned contact structures when analyzed with C‐AFM. Cells without resistive failure, in contrast, show consistent current levels.^[^ ^175^ ^]^ Although this example demonstrates C‐AFM as a diagnostic tool, the tip can also be used as a nano‐probe for detailed I‐V measurements, providing a more thorough analysis of individual contacts.

In addition to defect identification, AFM can be used for simultaneous in‐situ imaging and nanolithography by applying external stimuli, such as mechanical forces or thermal heating, through the AFM tip. As shown in Figure 9c, metal electrodes are precisely patterned on MoS_2_ using t‐SPL, avoiding common challenges of traditional lithography, such as photoresist contamination and material damage. This process utilizes a two‐layer polymer stack, where the top polymer is thermally decomposed by the heated probe, enabling residue‐free patterning and selective exposure for subsequent metal deposition.^[^ ^176^ ^]^

It is also evident that AFM‐based methods continue to evolve, with a clear trend toward pushing spatial resolution limits. While conventional AM‐ and FM‐KPFM techniques are still widely used for mapping surface potential and work functions, the recently developed pulsed force KPFM (PF‐KPFM) has achieved spatial resolutions below 10 nm. PF‐KPFM improves upon traditional methods by measuring the Coulomb force between the AFM tip and the sample based on intrinsic Fermi‐level alignment, thereby eliminating the need for the oscillating voltage required in conventional KPFM.^[^ ^177^ ^]^ Figure 9d illustrates surface potential maps obtained using PF‐KPFM, revealing the ohmic nature of the Al/Si interface, where band bending occurs, compared to the non‐ohmic contact in Si/Au, where a barrier to electron flow is formed.

### 6.3 Ceramics

Ceramics exhibit mechanical, electromechanical, and surface properties critical to their structural, functional, and biomedical applications. Parameters such as the elastic modulus, polarization behavior, and surface charge can be investigated using the AFM to investigate these properties through various measurement modes.

**Figure 10:**
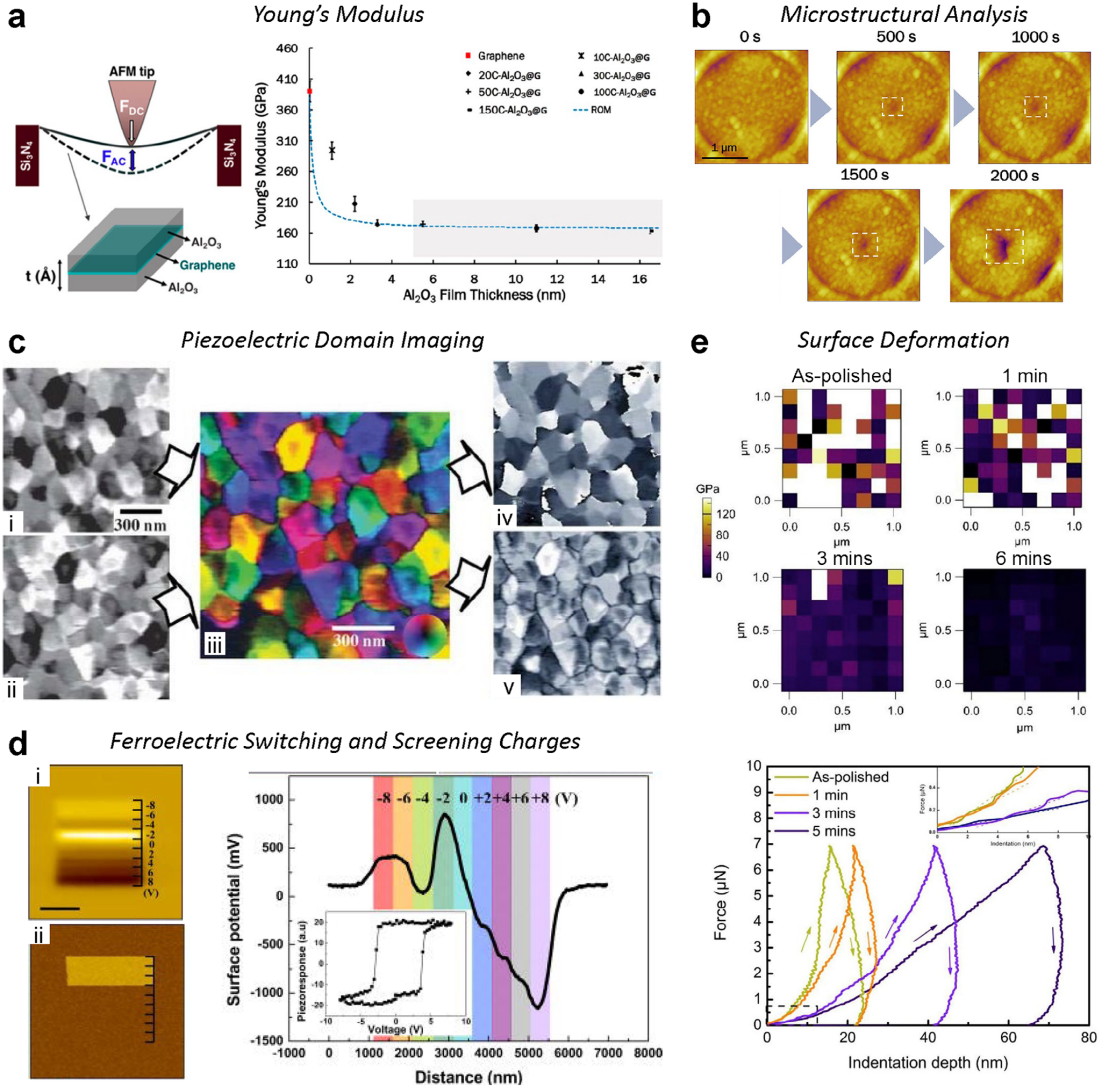
Applications of AFM in Ceramics. a) AFM deflection testing to measure the Young's modulus of suspended Al_2_O_3_‐graphene nanolayers. The elastic modulus transitions from graphene‐dominated stiffness to bulk alumina behavior with increasing thickness. b) Topography images showing progressive plastic damage in Al_2_O_3_‐graphene after static loading. Images a and b are reproduced with permission.^[^ ^179^ ^]^ Copyright 2020, American Chemical Society. c) Vertical (i) and lateral (ii) A_1ω_cosφ PFM images of PbTiO_3_ are combined to create a 2D vector map of the electromechanical response (iii). The color wheel legend represents the magnitude and direction of the response vectors, while scalar representations are provided through the angle (iv) and magnitude (v) images of the vectors. Reproduced with permission.^[^ ^26^ ^]^ Copyright 2006, Oxford University Press. d) KPFM surface potential (i) and PFM phase (ii) images of PbTiO_3_ films, showing a region scanned with varying applied voltage biases for each marked rectangular area. A surface potential line profile corresponding to (i) is displayed on the right, with an inset showing the piezoresponse hysteresis loop. Reproduced with permission.^[^ ^182^ ^]^ Copyright 2009, AIP Publishing. e) Variation in the human tooth enamel's elastic modulus with different immersion times in Coca‐Cola. Typical indentation curves for each state, used to calculate the elastic modulus, are shown below. Reproduced with permission.^[^ ^184^ ^]^ Copyright 2020, Elsevier.

As with other types of materials, AFM facilitates the measurement of mechanical properties in ceramics through nanoindentation techniques, where the tip interacts with the material surface to generate force‐deflection curves. These curves are analyzed using contact mechanics models such as Hertz, Oliver‐Pharr, or DMT to determine properties like Young's modulus or adhesion.^[^ ^178^ ^]^ **Figure** **10**a demonstrates an approach where the elastic modulus of Al_2_O_3_ nanolayers is measured free of substrate effects using a suspended setup on a holey transmission electron microscopy (TEM) grid.^[^ ^179^ ^]^ The results reveal a thickness‐dependent transition from graphene‐dominated stiffness to bulk alumina behavior, highlighting the precision of AFM in nanomechanical characterization.

In addition to elastic properties, specific setups of the AFM also enable the investigation of plastic properties and material strength through failure testing.^[^ ^180^ ^]^ Static dwelling tests apply constant loads on the sample surface by the tip point, simulating creep conditions. Figure 10b shows the progressive plastic damage that occurred with such conditions on Al_2_O_3_ surfaces, monitored through continuous topography scans.^[^ ^179^ ^]^ Modified AFM techniques may also incorporate cyclic mechanical loading through oscillating cantilevers, enabling fatigue testing and dynamic analyses.^[^ ^181^ ^]^

For functional ceramics, particularly piezoelectric and ferroelectric materials, AFM reveals stimulus‐dependent responses through PFM. Figure 10c presents PFM mapping of vertical (out‐of‐plane) and lateral (in‐plane) piezoresponse components in PbTiO_3_, combined into a quantitative 2D vector map of electromechanical response. This visualization reveals polarization orientations within the individual grains and response variations between grain centers and boundaries.^[^ ^26^ ^]^

Ferroelectric materials exhibit spontaneous polarization with associated screening charge redistribution during switching effects. Figure 10d demonstrates the complementary use of PFM and KPFM to analyze surface potential from polarization and screening charges on epitaxial PbTiO_3_ surfaces.^[^ ^182^ ^]^ Applied voltage variation enables quantitative analysis of relative charge contributions, while the inset demonstrates local switching properties through piezoresponse loops, analogous to macroscopic polarization‐electric field (P‐E) hysteresis curves.

For bio‐ceramics, it may be interesting to investigate the degradation and surface evolution under physiological or environmental conditions using the AFM.^[^ ^183^, ^184^ ^]^ Figure 10e depicts a study where AFM was used to characterize the effects of Coca‐Cola immersion on dental enamel.^[^ ^184^ ^]^ Elastic modulus mapping and force‐indentation curves indicate softening of the surface as well as increasing indentation depths with prolonged exposure, providing a nanoscale perspective on dental erosion.

### 6.4 Polymers and Soft Materials

Distinguished by its electron beam‐free methodology, AFM offers an advantage in polymer and soft materials research compared to other visualization techniques. Its versatility to image in both dry and aqueous conditions across a broad temperature range enables the study of polymeric and soft materials under physiologically relevant environments.

**Figure 11:**
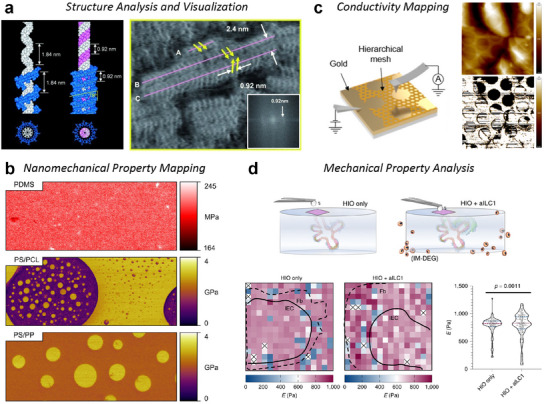
Applications of AFM in Polymers and Soft Materials. a) Double‐stranded and triple‐stranded helix models of an *it‐* and *st‐*PMMA stereocomplex. The AFM phase image identifies a mix of right‐handed (A and B) and left‐handed (C) helices, with the inset showing the Fourier transform of A. Observed dimensions inform the triple‐stranded model. Reproduced with permission.^[^ ^185^ ^]^ Copyright 2007, Wiley‐VCH GmbH. b) Bimodal AM‐FM modulus maps on materials with varying stiffness. Reproduced with permission.^[^ ^186^ ^]^ Copyright 2017, American Chemical Society. c) Schematic illustration of C‐AFM conducted on a hierarchical Pd mesh created through block copolymer templating. The corresponding topography and conductivity maps demonstrate the electrical pathways enabled by the interconnected structures. Reproduced with permission.^[^ ^187^ ^]^ Copyright 2022, American Chemical Society. d) Stiffness mapping of cell‐laden hydrogels using bead‐functionalized cantilevers, showing the experimental setup schematic, representative stiffness maps with and without immune cell incorporation, and statistical analysis of elastic modulus distributions. Reproduced with permission.^[^ ^188^ ^]^ Copyright 2021, Springer Nature.

Molecular visualization remains a central challenge in polymer research. By employing the non‐contact or tapping mode in AFM, the structure of such soft materials can be imaged with minimal damage. The phase delay in cantilever oscillation—arising from viscoelastic interactions with the sample—produces phase images with higher contrast compared to height images, facilitating high‐resolution analysis. For instance, in a poly(methyl methacrylate) (PMMA) stereocomplex study, AFM was used to elucidate the molecular architecture.^[^ ^185^ ^]^ While an earlier double‐stranded helical model had been previously proposed, AFM phase images revealed right‐handed and left‐handed helices with a reduced helical pitch for the outer *st‐*PMMA helix. This evidence led to a revised triple‐stranded helical model consistent with the observed stoichiometry and nanoscale dimensions (**Figure** **11**a), a level of detail unattainable with conventional XRD techniques.

Beyond structural imaging, the AM‐FM technique enables quantitative property mapping over a wide modulus range by simultaneously exciting the cantilever at two resonant frequencies.^[^ ^186^ ^]^ While based on the classic tapping mode, the dual‐frequency approach not only enables measurement of elastic moduli in homogeneous materials but also reveals mechanical contrasts that are difficult to detect in heterogeneous mixtures, as shown in the cases for polystyrene/polycaprolactone (PS/PCL) and polystyrene/polypropylene (PS/PP) polymer blends (Figure 11b).^[^ ^186^ ^]^

Building on the mentioned capabilities, electrical conductivity mapping at the nanoscale also becomes particularly relevant for both conductive polymers and polymer templates used in pattern transfer for conductive materials. Block copolymer self‐assembly, in particular, provides a facile route toward highly ordered nanopatterns, which can be utilized for creating functional electronic structures. As demonstrated in Figure 11c, C‐AFM measurements reveal the electrical pathways in hierarchically patterned metal meshes created through block copolymer templating, where the interconnected structure maintains conductivity across the entire network without requiring an underlying conduction layer.^[^ ^187^ ^]^

AFM‐based mechanical characterization is particularly valuable for analyzing hydrogels, which have gained attention in biological applications due to their tissue‐like properties and tunable mechanical characteristics. Tip modifications to nanometer or micrometer‐sized beads have improved measurement accuracy for such soft materials by mitigating damage and providing more reliable contact mechanics. As demonstrated in Figure 11d, bead‐functionalized cantilevers enable the mapping of local mechanical properties in cell‐laden hydrogels. The study revealed the roles of innate lymphoid cells in driving spatially heterogeneous matrix remodeling through coordinated stiffening and softening processes.^[^ ^188^ ^]^

### 6.5 Biological Materials

The complexity of biological materials requires tools that can probe their structure, dynamics, and function across multiple scales. AFM enables the investigation of these systems in their native states while also providing nanoscale maps of their physical properties at the molecular level. Integrating multiple measurement modes, combined with advances in instrumental precision and stability, has established AFM as a fundamental technique for quantifying structure‐function relationships in biological systems.

**Figure 12:**
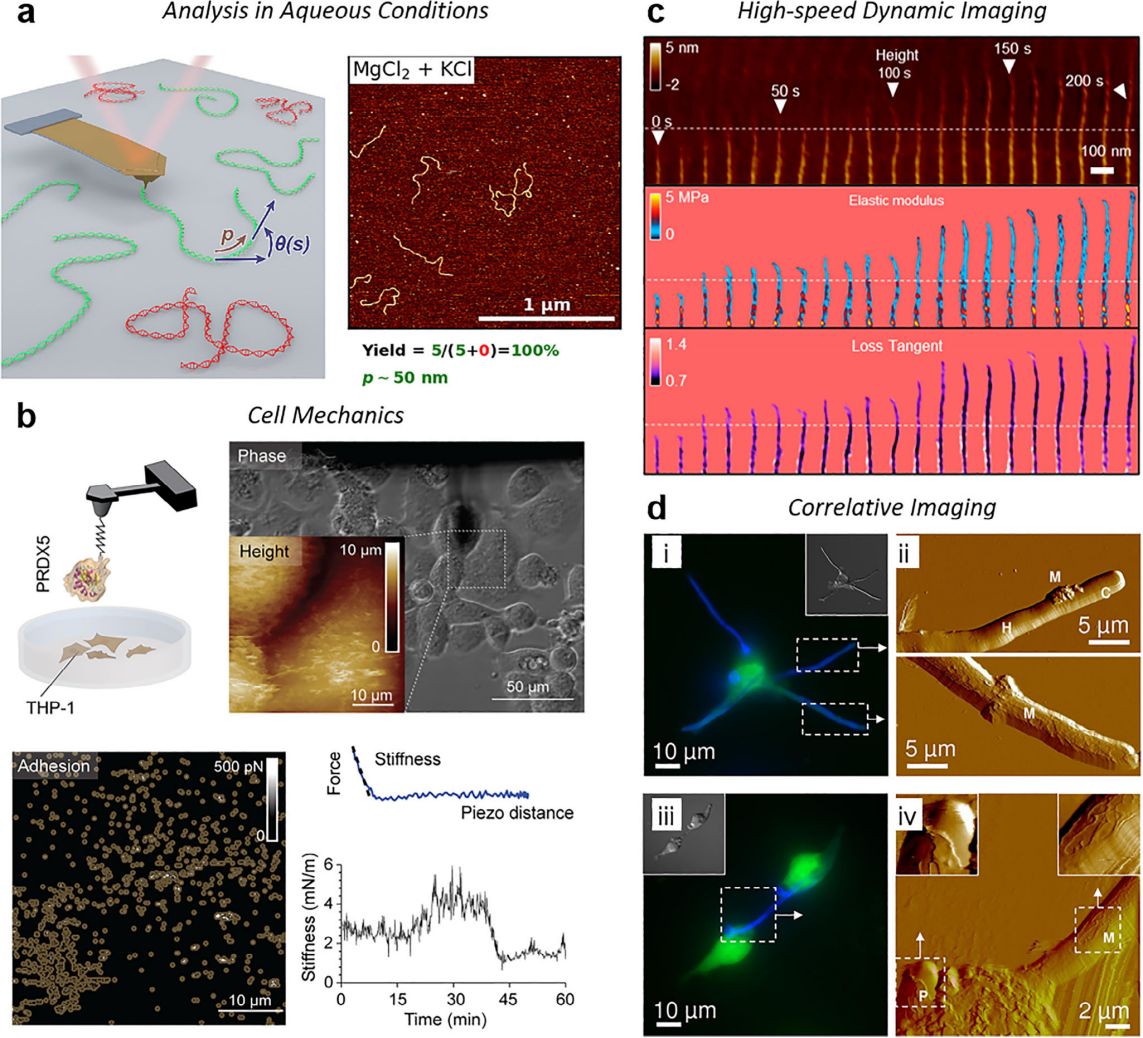
Applications of AFM in Biological Materials. a) Schematic and experimental DNA configurations imaged by AFM in liquid showing extended, equilibrated states (green) versus compact, kinetically trapped states (red). AFM image demonstrates DNA equilibration under physiological ionic conditions, yielding correct persistence length (p) and high yield of extended conformations. Reproduced with permission.^[^ ^189^ ^]^ Copyright 2019, American Chemical Society. b) Force‐distance based molecular interaction analysis of immune cells using protein functionalized tips. Height image (inset) and corresponding adhesion map identify specific binding events, while stiffness measurements track the cellular mechanoresponse to molecular recognition. Reproduced with permission.^[^ ^190^ ^]^ Copyright 2018, Elsevier. c) Collagen fibril growth dynamics visualized with high‐speed bimodal AFM, showing structural assembly stages in kymographs. Reproduced with permission.^[^ ^191^ ^]^ Copyright 2021, American Chemical Society. Licensed under CC BY 4.0. d) Correlative fluorescence and AFM deflection images of macrophage (green)‐*C. albicans* fungal pathogen (blue) interactions. AFM deflection images reveal characteristic surface features including macrophage membranes (M), fungal hyphae (H), and phagocytic cups (P). C indicates tip convolution artifacts. Reproduced with permission.^[^ ^192^ ^]^ Copyright 2012, American Chemical Society.

A key advantage of AFM regarding biological materials is its ability to operate in liquid environments under physiological conditions, though careful optimization of experimental parameters is essential to preserve native properties. For instance, recent work demonstrated successful imaging of equilibrated DNA molecules through precise control of ionic conditions.^[^ ^189^ ^]^ Using a MgCl_2_/KCl buffer system, researchers achieved high‐fidelity DNA imaging that yielded both the correct persistence length (∼50 nm, a measure of DNA's mechanical rigidity) and a high yield of analyzable molecules (**Figure** **12**a).^[^ ^189^ ^]^ Such configurations precisely characterized DNA's intrinsic mechanical properties in physiologically relevant conditions.

Force‐distance curve‐based AFM measurements using specifically modified probes further enhance surface mapping by simultaneously measuring molecular interactions. Using protein‐functionalized tips, AFM can map specific binding events on living cells while monitoring their mechanical responses. As shown in Figure 12b, this approach generated parametric maps of interaction forces that can be directly correlated with topographical features. It was also possible for the authors to track in situ changes in cell stiffness induced by molecular recognition events, with stiffness values extracted from the slope of approach curves in the contact region.^[^ ^190^ ^]^

The study of cellular mechanics and dynamics remains a growing area of interest, particularly in observing processes such as structural assembly. HS‐AFM enables real‐time observation of these dynamics, while recent developments in bimodal AFM allow simultaneous mapping of topography and mechanical properties at frame rates up to 5 fps.^[^ ^191^ ^]^ This capability has revealed previously unresolved stages of structural assembly, as demonstrated in studies of collagen fibrillogenesis using high‐speed bimodal AFM (Figure 12c).^[^ ^191^ ^]^

When combined with complementary techniques such as fluorescence or confocal microscopy, AFM achieves nanoscale resolution imaging and correlation of complex cellular structures. While fluorescence imaging provides structural labeling and identifies specific components through staining, its resolution is limited by the wavelength of light. Correlated fluorescence‐AFM addresses this limitation by pairing fluorescence's labeling capability with AFM's high‐resolution topography. For example, Figure 12d shows two cases of macrophage‐fungal pathogen interactions.^[^ ^192^ ^]^ In the upper set, AFM scans reveal the contrasting surface characteristics of the two fungal hyphae (branching filaments): smooth (unmodified hyphae) and rough (covered with macrophage membrane). The lower set shows two macrophages engulfing the same hypha, with AFM confirming the rough surface covered by the macrophage membrane, indicating internalization.

While the selected examples presented here demonstrate the use of AFM in biological contexts from molecular to cellular scales, these techniques have been widely applied across an even broader range of length scales, including complex and larger‐in‐size systems. For comprehensive overviews of AFM applications spanning molecular, cellular, and tissue‐level studies, readers are referred to the following sources.^[^ ^9^, ^193^, ^194^, ^195^ ^]^

### 6.6 Nanomaterials

By interfacing with materials at atomic‐level precision, AFM occupies a pioneering position in nanoscale research. Beyond electrical, mechanical, and chemical characterization, AFM has also driven advancements in developing methodologies for engineering applications, such as those in nanoelectronics, where the ability to manipulate and tune material behavior is a critical factor for innovation.

**Figure 13:**
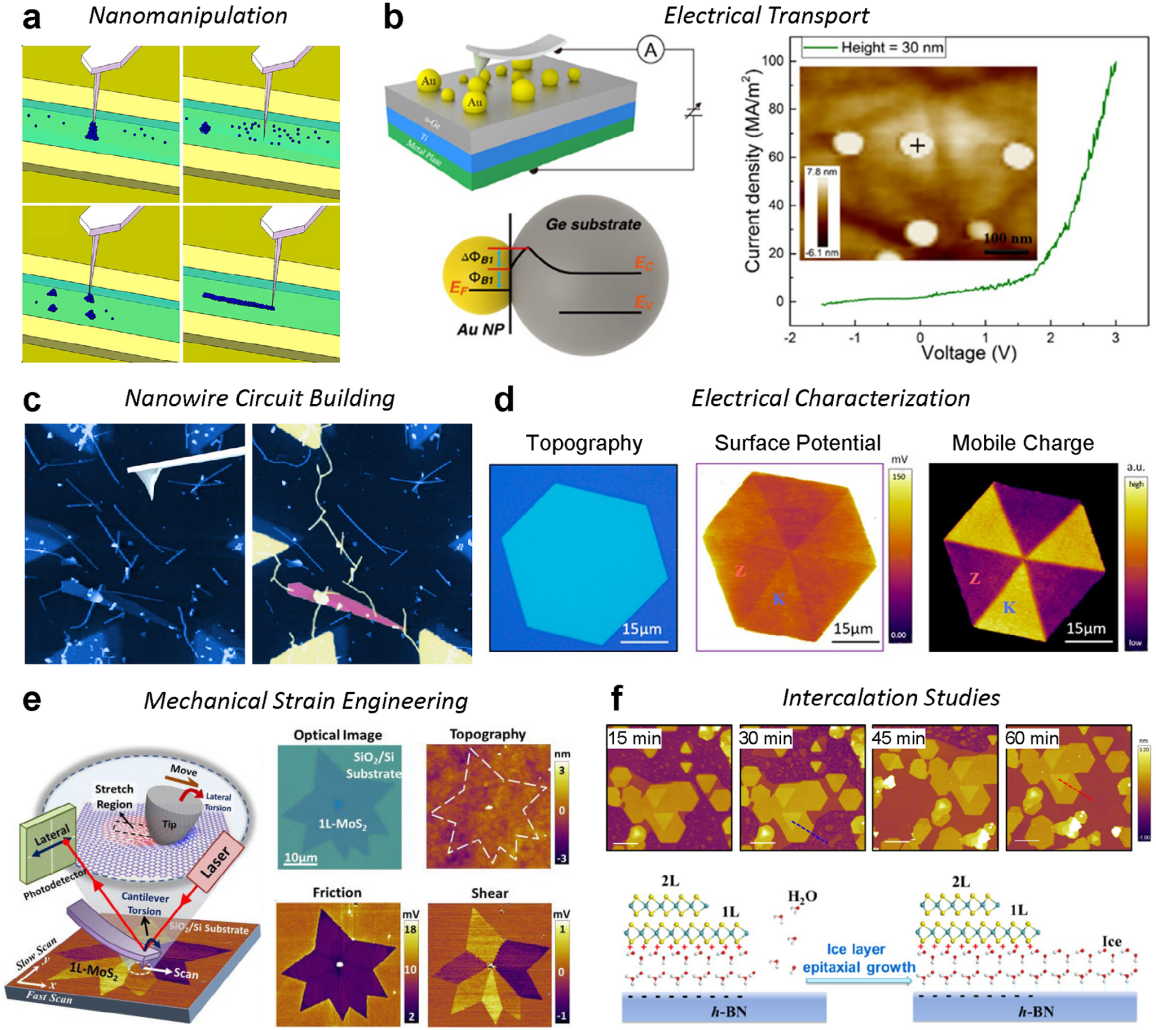
Applications of AFM in nanomaterials. a) Nanoparticle manipulation and assembly using AFM tip‐induced dielectrophoresis (DEP). Positive DEP forces enable the assembly into dots, arrays, or lines (counterclockwise from top left), while negative forces repel particles to prevent tip contamination (top right). Reproduced with permission.^[^ ^196^ ^]^ Copyright 2017, American Chemical Society. b) Schematic diagram illustrating C‐AFM measurements on Au NP/Ge diodes and the corresponding energy diagram of the Schottky junction. J‐V characteristics demonstrate rectifying behavior, where inset shows the topography image, with the cross marking the analyzed NP. Reproduced with permission.^[^ ^197^ ^]^ Copyright 2016, American Chemical Society. c) Dark‐field microscopy images before and after manipulating gold nanowires using the AFM tip to form an electrical connection between a graphene sample and microelectrodes, creating a four‐terminal device. Reproduced with permission.^[^ ^198^ ^]^ Copyright 2019, American Chemical Society. d) Optical topography of a hexagonal WS_2_ flake, with corresponding surface potential and mobile charge carrier density AFM images. The reduced mobile charge carrier density in the Zigzag (Z) regions compared to the Klein (K) regions indicates a larger local bandgap in the Z regions relative to the K. Reproduced with permission.^[^ ^199^ ^]^ Copyright 2020, American Chemical Society. e) Schematic of TSM, where friction drives stretch deformation, and TSM signals arise from the nonlinearity between stretch force and deformation direction. Experimental results are shown for a star‐shaped monolayer MoS_2_ flake. Reproduced with permission.^[^ ^200^ ^]^ Copyright 2019, AIP Publishing. f) AFM topography images showing the evolution of water intercalation at the NbS_2_/BN van der Waals heterostructure interface, along with a schematic diagram illustrating the intercalation process and the resulting structure. Reproduced with permission.^[^ ^201^ ^]^ Copyright 2019, IOP Publishing.

In AFM‐based material engineering, applying electrical or mechanical stimuli to the probe enables precise control of target properties and structural arrangements. As an example of positional control, tip‐induced dielectrophoresis (DEP) utilizes electric field gradients to assemble and organize nanoparticles into defined patterns such as dots, arrays, and lines.^[^ ^196^ ^]^ Positive DEP forces attract nanoparticles to facilitate assembly, while negative forces repel them, preventing tip contamination and maintaining operational integrity (**Figure** **13**a).

For nanomaterials with challenging geometries or nanoscale dimensions, C‐AFM offers an approach to studying current transport properties, overcoming the limitations of conventional macroscopic electrode configurations. Figure 13b illustrates C‐AFM measurements on gold nanoparticle (AuNP)/Ge Schottky diodes, where the conductive tip was precisely positioned at the apex of individual NPs, enabling localized current measurements. The rectifying current density‐voltage (J‐V) characteristics were examined, and the contact area was calculated based on the height of the AuNPs.^[^ ^197^ ^]^

Creating multiple electrical contacts on nanoscale materials further presents significant challenges, particularly for measurements that require complex configurations, such as four‐terminal setups that eliminate contact resistance effects. In the study shown in Figure 13c, AFM‐assisted nanowire circuit assembly enabled the creation of such circuits. In this work, gold nanowires were drop‐cast onto substrates and precisely rearranged using an AFM tip to create electrical connections between a graphene flake and pre‐deposited microelectrodes. This approach enables electrical characterization while maintaining measurement accuracy through multi‐terminal configurations for nano‐objects as small as tens of nanometers.^[^ ^198^ ^]^

AFM techniques have also become a common method to characterize the electrical properties of 2D materials, including surface potential, current distribution, and charge transport. Notably, multi‐frequency electrical measurement modes, such as dual‐harmonic EFM, have facilitated quantitative mapping of critical physical parameters such as carrier type and concentration. In the study shown in Figure 13d, KPFM and dual‐harmonic EFM were used to reveal nanoscale domains in monolayer WS_2_, with distinct local surface potentials and variations in mobile charge carrier density corresponding to local bandgap differences. These findings, supported by theoretical predictions and Raman mapping, underscore the utility of AFM in resolving electronic heterogeneities across scales spanning from atomic to mesoscopic levels.^[^ ^199^ ^]^

The ability to track torsional movements of the AFM tip expands its efficacy for investigating anisotropic behaviors and structural changes in 2D materials under mechanical deformation. For instance, torsional scanning mode (TSM) has revealed strain‐induced in‐plane anisotropic shear behaviors in monolayer MoS_2_, with distinct contrasts corresponding to crystallographic orientation (Figure 13e).^[^ ^200^ ^]^ During TSM measurements, the AFM tip scans parallel to the cantilever axis while recording lateral torsion. In flexible films weakly adhered to rigid substrates, localized puckering induces simultaneous relaxation at the leading edge and stretching at the trailing edge of the tip‐contact area. This effect was particularly evident in the star‐shaped monolayer MoS_2_ sample featured in Figure 13e, which showed pronounced contrasts in TSM (shear) imaging while remaining uniform in the corresponding FFM (friction), optical, and topographical images.

In addition to probing anisotropic mechanical behaviors, AFM has been used to investigate interfacial molecular interactions and transfer dynamics in 2D materials. Figure 13f presents a study on H_2_O intercalation behavior within the NbS_2_/BN heterostructure. Initial AFM images reveal that H_2_O molecules cluster around NbS_2_ regions, while some form discrete islands on the h‐BN surface. Over time, these molecules exhibit epitaxial growth from the intercalated layer, eventually forming a complete water overlayer on the h‐BN surface.^[^ ^201^ ^]^ Complementary KPFM and EFM measurements were used to analyze surface potential distributions and mobile charge carrier behavior, sharing methodological parallels to the study in Figure 13d. These findings demonstrate how the diverse operational modes in AFM can be strategically used to probe specific properties and dynamic processes in nanomaterials.

### 6.7 Composite and Hybrid Materials

The multifunctional and adaptable nature of composite materials arises from their complex microstructures, where molecular, interfacial, and dispersed phase interactions create emergent properties. Understanding these interactions at the component level is thus essential for advancing material development. Conventional characterization techniques, however, often provide only averaged macroscopic properties or lack sufficient spatial resolution. AFM overcomes these limitations by enabling component‐wise mapping of structural and functional attributes at nanoscale resolution. This section examines the applications of AFM in investigating composite and hybrid materials, with a focus on polymer composites and metal‐organic‐frameworks (MOFs).

**Figure 14:**
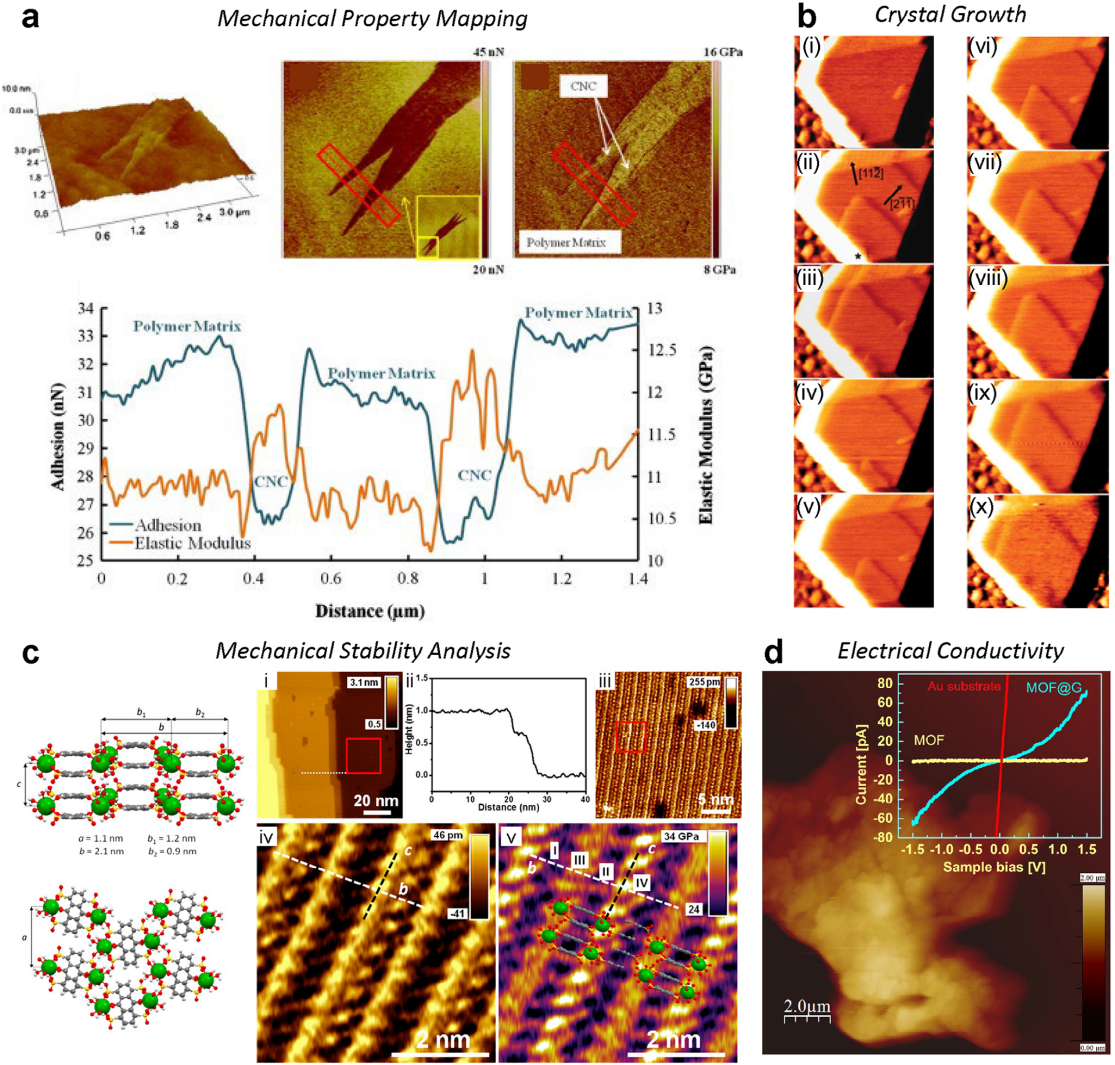
Applications of AFM in Composite and Hybrid Materials. a) 3D height image, adhesion map, and elastic modulus map of a cellulose nanocrystal (CNC) composite. Line profiles show the average adhesion and elastic modulus in the red‐boxed region. Reproduced with permission.^[^ ^202^ ^]^ Copyright 2012, Elsevier. b) In‐situ AFM deflection images of the {111} facet of a HKUST‐1 crystal, showing the progression of two‐dimensional crystal growth originating from a single nucleation site and spreading across the surface. Reproduced with permission.^[^ ^203^ ^]^ Copyright 2009, Royal Society of Chemistry. c) Side and top view schematic representations of the atomic structure of a cerium‐based MOF. Corresponding bimodal AFM results include: i) topographic image, ii) height profile along the dashed line in (i), iii) magnified view of the region marked in (i) with sub nanometer resolution, iv) further enlarged angstrom‐scale resolved image of the region marked in (iii), and v) elastic modulus map of the area shown in (iv). Reproduced with permission.^[^ ^204^ ^]^ Copyright 2017, American Chemical Society. Licensed under CC BY 4.0. d) Topographic AFM image showing the granular morphology of a MOF‐graphene (MOF@G) composite. The inset presents I‐V response curves, highlighting the significantly enhanced conductivity of the hybrid MOF@G compared to the pristine MOF. Reproduced with permission.^[^ ^205^ ^]^ Copyright 2019, Elsevier.

In polymer composites, AFM enables precise nanomechanical property mapping. A study on cellulose nanocrystal (CNC) composites demonstrated the technique's capability to measure adhesion and elastic modulus across the interphase region between cellulose and a poly(vinyl alcohol)‐poly(acrylic acid) polymer matrix.^[^ ^202^ ^]^ The analysis exposed a gradual transition in elastic modulus from the CNC interface to the surrounding matrix, attributed to a gradient in ester linkage density (**Figure** **14**a). Despite the valuable mechanical insights provided, such force mapping experiments can confront significant temporal constraints. Traditional force spectroscopy methods require approximately a few seconds per curve, rendering comprehensive high‐resolution maps a time‐intensive process that can extend from several hours to days. Technological advancements are progressively mitigating these acquisition speed limitations for more efficient and detailed mechanical characterizations.

MOFs exemplify complex material systems where AFM is extensively employed to characterize crystal growth, mechanical stability, and electric behavior. In particular, understanding the crystallization behavior of these crystalline, nano‐porous materials is essential, as it directly impacts their performance in catalytic, adsorption, and ion‐exchange processes. Moreover, controlling the morphology of nano‐ or micron‐sized MOF crystals requires detailed insights into their growth mechanisms. For example, during an in‐situ AFM investigation of HKUST‐1 (MOF‐199) crystallization, sequential imaging of the {111} facet revealed a two‐dimensional growth mechanism.^[^ ^203^ ^]^ The observations revealed that the crystal expansion occurs via layer‐by‐layer nucleation, initiating from a single site and subsequently propagating across the surface (Figure 14b).

Methods for characterizing the mechanical properties of MOFs can be broadly categorized into nanoindentation‐based techniques and multifrequency AFM operations. The nanoindentation approach, illustrated in Figure 14a, involves repeated cycles of tip approach, interaction recording, and retraction to measure mechanical responses. In contrast, bimodal AFM, previously discussed in Figures 11b and 12c, utilizes AM‐FM feedback to rapidly and precisely map nanomechanical properties. For example, in the assessment of mechanical stability, bimodal AFM was employed to generate sub‐nanometer‐resolved elastic modulus maps on the surface of a Ce‐based MOF (Figure 14c). These maps, correlated with the atomic structure, revealed regions of mechanical heterogeneity linked to variations in the MOF's crystallographic structure.^[^ ^204^ ^]^

While MOFs are typically insulating due to their organic linker components and wide band gaps, the development of conductive MOFs has attracted attention for their potential in electronic and energy applications. Analyzing the electrical characteristics of conductive MOFs provides a foundation for understanding and optimizing their performance in devices. In one study, C‐AFM was used to evaluate the electrical conductivity of MOF‐graphene (MOF@G) composites derived from an insulating Zr‐based MOF.^[^ ^205^ ^]^ The findings revealed a composite conductivity enhancement attributed to charge delocalization at the MOF@G interface, compared to the pristine MOF (Figure 14d).

### 6.8 Energy Materials

Energy materials, such as those used in batteries, fuel cells, and photovoltaics, often rely on finely tuned material interfaces and nanoscale features to achieve optimal performance. In this context, AFM can resolve topographical features and material interfaces critical for charge transport and ion diffusion. Advanced AFM techniques such as C‐AFM and KPFM enable mapping electrical properties like conductivity and contact potential differences (CPD). ESM, in particular, can specifically analyze the electrochemical properties of battery components by applying electrical signals to the probe to observe electrochemical reactions in real‐time.

**Figure 15:**
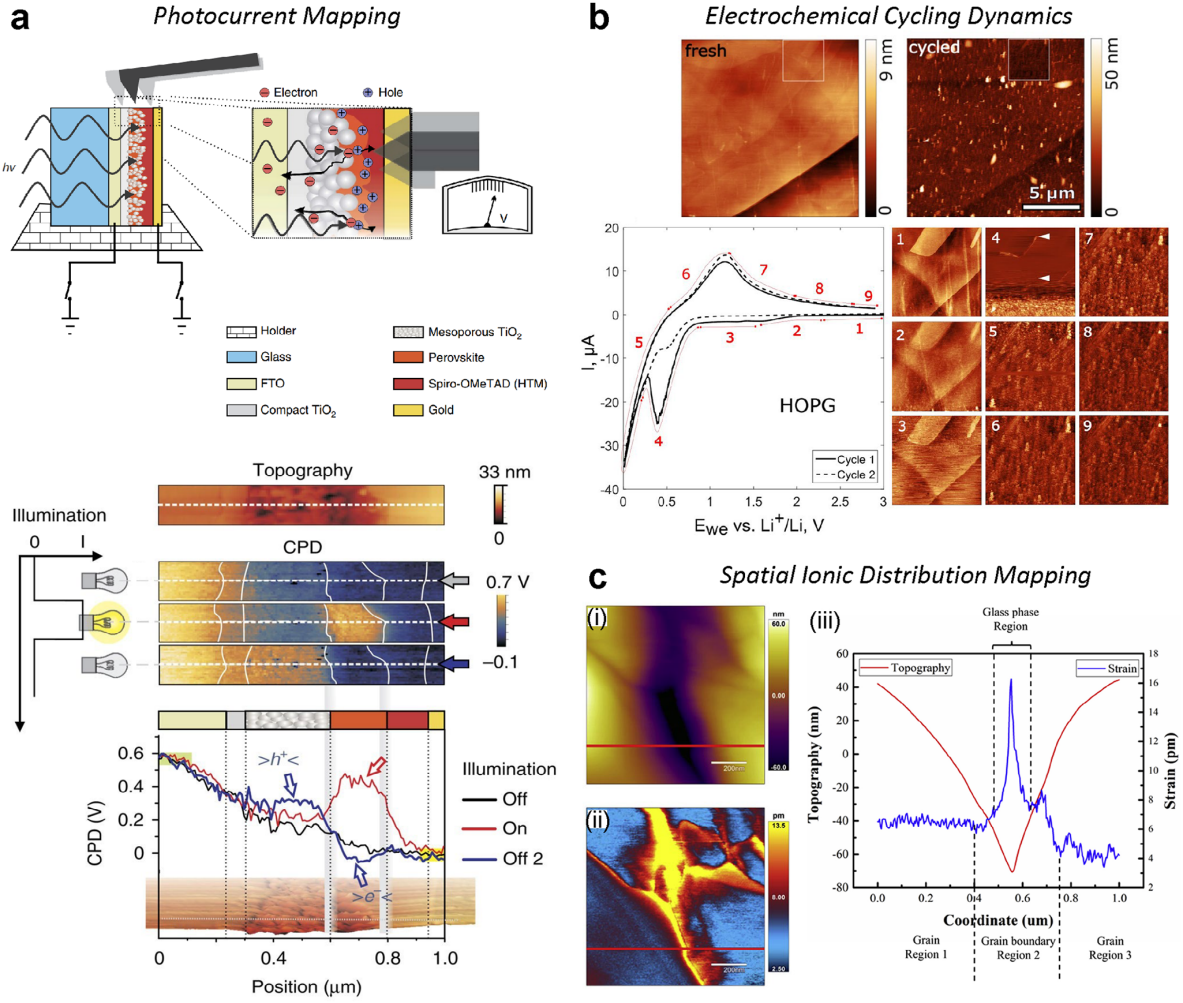
Applications of AFM in Energy Materials. a) Real‐space visualization of charge transport in a lead halide perovskite solar cell using KPFM. (Upper panel) Side and top view schematics illustrating the material interfaces and measurement principles. (Lower panel) Topography image, CPD maps, and line profiles under short‐circuit conditions: before illumination (black, Off), during illumination (red, On), and immediately after illumination (blue, Off 2). Reproduced with permission.^[^ ^206^ ^]^ Copyright 2014, Springer Nature. b) EC‐AFM topography showing the formation of the SEI on HOPG during cycling. The images capture the progression of surface morphology from the pristine state through successive cycling stages, with the white arrow indicating the onset of SEI nucleation. Reproduced with permission.^[^ ^207^ ^]^ Copyright 2020, Springer Nature. c) ESM images of a LAGP solid electrolyte, showing the surface topography (i), electrochemical strain map (ii), and corresponding line profiles revealing enhanced strain at the grain boundary due to differences in Li‐ion conductivity. Reproduced with permission.^[^ ^208^ ^]^ Copyright 2020, Elsevier.

An illustrative application of KPFM is mapping the CPD across the cross‐section of a lead halide perovskite solar cell under controlled illumination (**Figure** **15**a).^[^ ^206^ ^]^ Light is illuminated through the glass substrate, while open‐ and short‐circuit conditions are controlled by connecting the top and bottom electrodes, which are oriented perpendicular to the scanning plane of the AFM tip. In the dark (Off state), the CPD line profile shows a gradual decrease between the two electrodes, reflecting differences in the work function. Under illumination (On state), an increase in CPD is observed in the perovskite layer, indicating the presence of additional positive charge carriers. After the illumination is turned off (Off 2 state), the CPD distribution does not fully return to its initial state, suggesting that charge carriers may be trapped in material defects.

In lithium‐ion batteries, in‐situ AFM, or EC‐AFM, is commonly used to investigate the formation of the SEI. This technique involves applying electrochemical reactions directly to the material while simultaneously scanning its topography. The sample serves as the working electrode, while separate counter and reference electrodes are used with all components enclosed in a sealed cell filled with electrolyte. Consequently, the SEI layer formed on the electrode material surface during the cycling process can be analyzed in real‐time, as demonstrated by the results shown for an HOPG electrode in Figure 15b.^[^ ^207^ ^]^

While the previously mentioned ESM technique has been mainly utilized in the examination of lithium‐ion behavior in cathode materials, it has also been applied to characterize solid electrolytes used for all‐solid‐state battery systems, such as LAGP (Li_1.5_Al_0.5_Ge_1.5_(PO_4_)_3_). In this study, ESM correlated surface topography with electrochemical strain, identifying significant strain localization at grain boundaries due to variations in lithium‐ion conductivity (Figure 15c). The line analysis revealed a pronounced strain peak at the interface, underscoring the heterogeneity of ion transport as well as the need for AFM‐based spatial visualization and quantitative analysis within these materials.^[^ ^208^ ^]^

## 7 Closing Remarks and Outlook

The journey of AFM technology reflects a remarkable evolution from a surface imaging tool to an integrated materials characterization platform. As demonstrated throughout this review, AFM's expansion into diverse measurement modes and applications has been driven by continuous technological innovations and growing demands from various scientific fields. This evolution has naturally led to increasing integration in terms of both measurement capabilities and cross‐disciplinary applications.

The convergence of multiple characterization techniques within AFM has enabled unprecedented insights into material properties at the nanoscale. Recent breakthroughs exemplify this trend; electron spin resonance (ESR) integration with AFM has achieved single‐molecule spin detection for quantum computing applications,^[^ ^209^ ^]^ while tip‐enhanced Raman spectroscopy (TERS) enables molecular‐level chemical mapping for surface chemistry research.^[^ ^210^ ^]^ Most notably, quantum sensing through nitrogen‐vacancy (NV) defects in diamond tips has revolutionized magnetic imaging, achieving visualization of complex magnetic structures with atomic sensitivity.^[^ ^211^ ^]^ The development of three‐dimensional characterization methods, particularly when combined with techniques like SEM, has further expanded AFM's capabilities to reveal hidden material properties such as buried interfaces and domain structures.^[^ ^212^, ^213^ ^]^ Each of these advancements represents technical innovation that is successfully converged of multiple scientific disciplines, demonstrating how cross‐disciplinary collaboration continues to push the boundaries of materials characterization.

However, this technological sophistication has simultaneously created a growing expertise gap between AFM specialists and potential collaborators or users. As measurement modes have multiplied and data analysis has become increasingly complex, the risk of misinterpretation or experimental artifacts has similarly increased. What began as a relatively straightforward imaging technique now often requires specialized knowledge across multiple domains from nanomechanics to electronics and data science, presenting significant barriers to entry for non‐specialists.

As AFM measurements become increasingly refined while generating complex multidimensional datasets, the field naturally intersects with artificial intelligence and machine learning. This intersection is not merely a technological trend but a necessary evolution driven by the growing complexity of AFM data and the need for more efficient analysis methods. Integrating AI/ML with AFM presents opportunities and challenges that can only be addressed through meaningful collaboration between domain experts. AFM specialists bring a crucial understanding of measurement physics and instrumentation, materials scientists provide insights into physical phenomena and sample characteristics, and data scientists contribute expertise in advanced analysis methods. While AI approaches offer potential solutions to the expertise gap through automated analysis and user‐friendly interfaces, they also risk creating a new form of knowledge barrier if implemented without attention to interpretability and user education.

Looking ahead, the success of AFM technology will increasingly depend on the effective integration of diverse expertise. The development of automated systems and AI‐assisted measurements does not diminish the importance of domain knowledge; instead, it emphasizes the need for a deeper understanding of measurement principles and material properties. Future breakthroughs will likely emerge from the synergistic combination of advanced measurement techniques, intelligent data analysis, and most importantly, collaborative efforts that bridge different scientific disciplines. Current commercial systems already feature programmable functions, and future iterations will likely incorporate machine learning algorithms for real‐time experimental optimization. The multidimensional nature of AFM data particularly suits these advances, as computer vision and deep learning techniques can extract complex material parameters from large datasets more efficiently than traditional analysis methods.

Addressing the growing expertise gap represents a critical direction for the field moving forward. This challenge requires technological solutions and methodological and educational approaches. Standardized protocols, improved user interfaces, systematic training modules, and clearer guidelines for data interpretation will be essential to ensure that advanced AFM techniques remain accessible beyond specialist circles. Without such efforts, the increasing sophistication of AFM risks limiting its impact rather than expanding it.

This outlook suggests that while AFM continues to advance, its most significant developments will come from the convergence of different fields and expertise. The future of AFM lies not just in new measurement capabilities or automated analysis but in the effective collaboration between experts who can bridge the gap between traditional AFM measurements, advanced analytical techniques, and modern computational methods. As systems become more complex and data more abundant, combining domain knowledge from different fields will become increasingly crucial for pushing the boundaries of materials characterization and discovery.

## Author Contributions

S.J., S.E., Y.C., U.J., Y.C., W.Y., and K.P. contributed equally to this work. Soyun Joo: Writing–Section 6, Conceptualization. Seongmun Eom: Writing–Sections 1 and 7. Youngwoo Choi: Writing–Section 4. Uichang Jeong: Writing–Section 3. Yoonhan Cho: Writing–Section 3. WonJeong Yu: Writing–Section 5, Conceptualization–ToC Graphic. Kunwoo Park: Writing–Section 2. Seungbum Hong: Writing–review & editing, Supervision, Project administration, Funding acquisition.

## Conflict of Interest

The authors declare no conflict of interest.
